# The ZO-1 protein Polychaetoid as an upstream regulator of the Hippo pathway in *Drosophila*

**DOI:** 10.1371/journal.pgen.1009894

**Published:** 2021-11-08

**Authors:** Qingliang Sang, Gang Wang, David B. Morton, Hui Wu, Baotong Xie

**Affiliations:** Integrative Biomedical and Diagnostic Sciences Department, School of Dentistry, Oregon Health and Science University, Portland, Oregon, United States of America; New York University, UNITED STATES

## Abstract

The generation of a diversity of photoreceptor (PR) subtypes with different spectral sensitivities is essential for color vision in animals. In the *Drosophila* eye, the Hippo pathway has been implicated in blue- and green-sensitive PR subtype fate specification. Specifically, Hippo pathway activation promotes green-sensitive PR fate at the expense of blue-sensitive PRs. Here, using a sensitized triple heterozygote-based genetic screening approach, we report the identification of the single *Drosophila* zonula occludens-1 (ZO-1) protein Polychaetoid (Pyd) as a new regulator of the Hippo pathway during the blue- and green-sensitive PR subtype binary fate choice. We demonstrate that Pyd acts upstream of the core components and the upstream regulator Pez in the Hippo pathway. Furthermore, We found that Pyd represses the activity of Su(dx), a E3 ligase that negatively regulates Pez and can physically interact with Pyd, during PR subtype fate specification. Together, our results identify a new mechanism underlying the Hippo signaling pathway in post-mitotic neuronal fate specification.

## Author summary

The Hippo signaling pathway was originally discovered for its critical role in tissue growth and organ size control. Its evolutionarily conserved roles in various biological processes, including cell differentiation, stem cell regeneration and homeostasis, innate immune biology, as well as tumorigenesis, have been subsequently found in other species. During the development of the *Drosophila* eye, the Hippo pathway promotes green- and represses blue-sensitive photoreceptor (PR) subtype fate specification. Taking advantage of this binary PR fate choice, we screened *Drosophila* chromosomal deficiency lines to seek new regulators of the Hippo signaling pathway. We identified the *Drosophila* membrane-associated ZO-1 protein Pyd as an upstream regulator of the Hippo pathway to specify PR subtypes. Our results have demonstrated that Pyd represses Su(dx)’s activity in the Hippo pathway to specify PR subtypes. Our results demonstrate a new mechanism underlying the Hippo signaling pathway in post-mitotic neuronal fate specification.

## Introduction

Generating neuronal diversity during the development of a sensory organ is a prerequisite for the organ to perceive and discriminate various external stimuli. For example, the perception of color relies on comparing the outputs of multiple light-sensing photoreceptor (PR) subtypes with different spectral sensitivities [1–3]. During development, the fate of sensory neurons is progressively restricted toward terminal differentiation, finally generating diverse neuronal subtypes. Although the role of transcriptional regulations during neuronal terminal differentiation has been extensively studied [4,5], the details of how specific signaling pathways influence this process are not well understood. Here we use the blue- and green-sensitive PR binary fate decisions in the *Drosophila* eye as a model to understand the role of the Hippo pathway in post-mitotic neuronal terminal differentiation.

**Fig 1:**
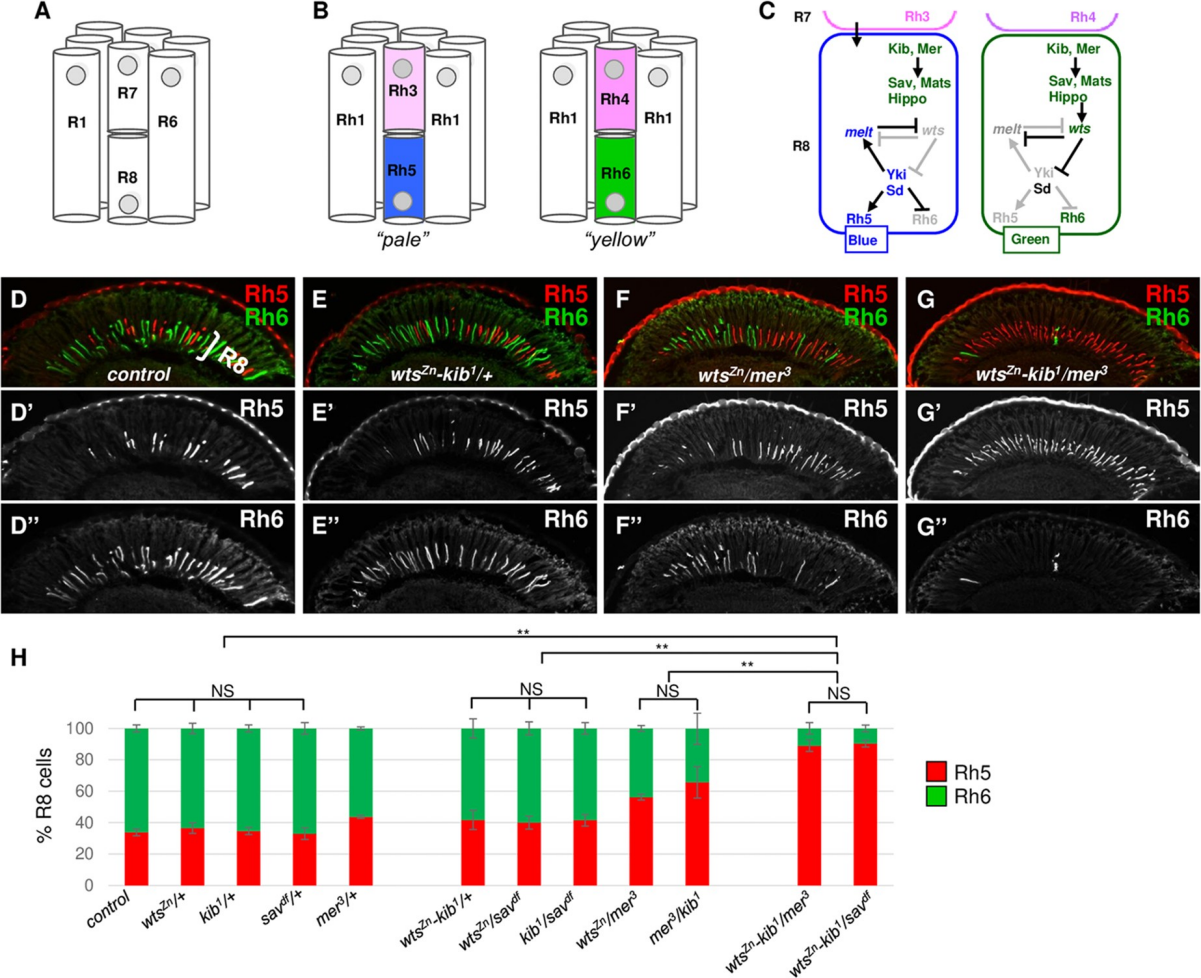
Tripe heterozygote-based phenotype enhancement assays for R8 subtypes. (A) Schematic of an adult ommatidium with six outer photoreceptors (PRs) (R1-R6) and two inner PRs (R7 and R8). (B) Two main, pale (p) and yellow (y), ommatidial subtypes and corresponding Rhodopsin (Rh) expression patterns. (C) The Hippo pathway and *melt* specify yR8 (Wts+, Rh6+) vs. pR8 (Melt+, Rh5+) fates. pR7s send instructive pR7-to-pR8 signals (arrow from pink pR7) to activate *melt* in yR8s, and Melt, together with Yki, represses *wts* expression. In yR8s, Hippo signaling suppresses Yki activity and represses *melt* expression. Genes or proteins that are inactive or not expressed are denoted by grey font, while those that are expressed and/or active are represented by blue, green or black font. (D-G) Adult eye cryosections stained for Rh5 (red) and Rh6 (green) from control (D), *wts*^*Zn*^ and *kib*^*1*^ heterozygous (E), *wts*^*Zn*^ and *mer*^*3*^ heterozygous (F), or *wts*^*Zn*^, *kib*^*1*^ and *mer*^*3*^ triple heterozygous (G) flies. (H) Quantification of Rh5- and Rh6-positive R8s in control flies as well as flies with Hippo pathway heterozygous, double heterozygous or triple heterozygous mutations. Graph presents proportion of R8s (y axis) that express Rh5 (red) or Rh6 (green). Two-tailed, unpaired t test. NS: not significant. **P < 0.001. Error bars represent standard deviation. control: n = 8 retinas, n = 1986 R8s; *wts*^*Zn*^/+: n = 8 retinas, n = 2036 R8s; *kib*^*1*^*/+*: n = 8 retinas, n = 1796 R8s; *sav*^*df*^*/+*: n = 7 retinas, n = 1709 R8s; *mer*^*3*^*/+*: n = 6 retinas, n = 1488 R8s; *wts*^*Zn*^-*kib*^*1*^/+: n = 8 retinas, n = 1867 R8s; *wts*^*Zn*^/*sav*^*df*^: n = 8 retinas, n = 2001 R8s; *kib*^*1*^/*sav*^*df*^: n = 8 retinas, n = 1681 R8s; *wts*^*Zn*^/*mer*^*3*^: n = 6 retinas, n = 1421 R8s; *mer*^*3*^/*kib*^*1*^: n = 6 retinas, n = 1349 R8s; *wts*^*Zn*^-*kib*^*1*^/*mer*^*3*^: n = 6 retinas, n = 1421 R8s; *wts*^*Zn*^-*kib*^*1*^/*sav*^*df*^: n = 6 retinas, n = 1356 R8s. Refer to Supporting Information S1 Text for detail genotypes. Also see S1 Fig.

The *Drosophila* eye is a powerful model to understand the principles of neuronal development [1,6–8]. The *Drosophila* compound eye contains ~750 individual units, ommatidia, each of which consists of eight PRs: the outer PRs R1-R6 and the inner PRs R7-R8 (**Fig 1A**). There are two main subtypes of ommatidia: pale (p) and yellow (y) ommatidia, present in the adult *Drosophila* eye (**Fig 1B**). The outer photoreceptors R1-R6 in both p and y ommatidia express the broad spectrum light sensitive opsin Rhodopsin 1 (Rh1) and are responsible for dim light vision and motion detection. However, the inner R7 and R8 cells express Rhodopsins with different spectral sensitivities, making them capable of performing color vision [9]. In p ommatidia, R7s express UV-sensitive Rh3 and R8s express blue-sensitive Rh5, while in y ommatidia, R7s express UV sensitive Rh4 and R8s express green-sensitive Rh6 (**Fig 1B**). The p and y subtypes are randomly distributed throughout the retina in roughly a 35:65 p:y ratio [10]. The p vs. y fate decision is first made in R7s via the stochastic activation of the transcription factor Spineless in yR7s during mid-pupation [11]. R7s that do not express Spineless (*i*.*e*. the pR7s) instruct their underlying R8s to adopt pale R8 (pR8) fate through Activin and BMP signaling [12]. R8s that do not receive the pR7 signals default to yellow R8 (yR8) fate [13,14]. The effectors involved in p vs. y R8 fate in R8s involve two proteins—Melted (Melt) and Warts (Wts) [15] (**Fig 1C**). *melt* encodes a pleckstrin homology domain-containing protein [16], while *wts* encodes a serine/threonine kinase that is a core component in the Hippo signaling pathway [17–19]. *melt* expression is activated in pR8s by the pR7-driven Activin and BMP signals and its expression leads to the transcriptional repression of *wts*. Conversely, *wts* represses *melt* expression in yR8s by suppressing the activity of the transcriptional coactivator Yorkie (Yki), the downstream effector of the Hippo pathway. Yki is necessary for *melt* expression in pR8s. Therefore, *wts*, *melt* and Yki form a double negative regulatory loop to ensure pR8 vs. yR8 subtype fate decision (**Fig 1C**) [10]. Yki, together with its DNA-binding partner Scalloped (Sd), regulates the output of the regulatory loop to promote the expression of blue-sensitive Rh5 and prevent the expression of green-sensitive Rh6 [20].

The Hippo pathway was originally discovered in *Drosophila* for its pivotal roles in tissue growth and organ size control [21]. Its critical and conserved roles in mammals have been subsequently identified in a wide range of biological processes, including stem cell regeneration and homeostasis, innate immune biology, cell differentiation, as well as tumorigenesis [19,22–24]. The components of the Hippo pathway can be classified into three categories: the core kinase complex, the downstream effectors and the upstream regulators. The core kinase complex contains the kinases Wts [17,18], Hippo (Hpo) [25–29], and Mob as tumor suppressor (Mats) [30], as well as the scaffold protein Salvador (Sav) [31,32]. Hpo phosphorylates Wts, affiliated by Sav and Mats, and Wts phosphorylates and inhibits the ability of Yki to enter the nucleus [33–35].

Multiple upstream inputs that feed into the core of the Hippo pathway in tissue growth have been identified in recent years. In *Drosophila*, these upstream inputs include the atypical cadherins Fat and Dachs [36–39], the cell adhesion molecule Echinoid (Ed) [40], the complex formed by the FERM-domain protein Expanded (Ex), Merlin (Mer) [41] and the WW-domain protein Kibra (Kib) [42,43], as well as the cell polarity determinants Crumbs (Crb), Lethal giant larvae (Lgl) and Scribble (Scrib) [44–47]. These upstream inputs act redundantly in tissue growth [41,42]. For example, growth defect in the imaginal disc carrying *kib* or *mer* mutations is much weaker compared to those carrying the mutations of the core components of the Hippo pathway. In contrast, double mutations for *ex* and *mer* or *kib* cause severe growth phenotype as demonstrated in *hpo* or *wts* mutations [41,42,48]. Interestingly, among these upstream regulators, *mer*, *kib* and *lgl* regulate the Hippo pathway in yR8 fate decision, while *fat*, *dachs* and *ex* are not involved in this process [49]. Additionally, *crb* is not required for the activation of the Hippo pathway during R8 subtype fate decisions [50]. Therefore, the pale and yellow binary fate assay in the *Drosophila* retina provides an efficient model with less upstream complication to understand the upstream regulation of the Hippo pathway.

Taking advantage of the pR8 and yR8 binary fate assay, we generated and carried out a sensitive and efficient genome-wide screening to identify the new regulators of the Hippo pathway. We identified the *Drosophila* ZO-1 protein Pyd as a new upstream regulator of the Hippo pathway. Using loss- and gain-of-function studies, we show that Pyd is required and sufficient to promote green-sensitive yR8 fate and repress blue-sensitive pR8 fate. We additionally determined the roles in PR subtype fate specification for *pez* and *suppressor of deltex* (*su(dx)*), the upstream regulators of the Hippo pathway in the *Drosophila* midgut epithelium [51,52]. Using epistasis analyses, we revealed that Pyd acts upstream of the core components and the upstream regulator Pez in the Hippo pathway, while it may function in parallel to Kib to repress Su(dx)’s activity to specify R8 subtypes. Together, our study identifies a new upstream regulator of the Hippo pathway that functions in post-mitotic neuronal fate specification.

## Results

### A triple heterozygote-based screening to identify new regulators of the Hippo pathway

A complementation test is generally used to determine whether or not two mutations define the same or different genes [53]. In most cases, if two recessive mutations that cause similar phenotypes are not in the same genes, the two mutations can be complemented by the corresponding wild type alleles in the F1 double heterozygotes and therefore manifest the wild type phenotype. However, when these mutations are in the genes of the same signaling pathway, they can fail to complement each other. Instead, the double heterozygote can exhibit a mutant phenotype [53,54]. To test whether this non-complementation between the genes in the same pathway can be used to screen the *Drosophila* deficiency collections [55,56] to identify new components of the Hippo signaling pathway, we performed complementation assays for pR8 and yR8 subtype fate specification between a mutation of *wts*, the core component of the Hippo pathway, and mutations in other genes in the pathway. Compared to the ratios of pR8 (Rh5+: 33.9±2.2%) and yR8 (Rh6+: 66.1±2.2%) in wild type flies (**Fig 1D and 1H**), flies heterozygous for a hypomorphic *wts* enhancer trap line *wts*^*Zn*^ [15] (*wts*^*Zn*^/+) (Rh5+: 36.5±3.4%, Rh6+: 63.5±3.4%), a null *kib* allele *kib*^*1*^ [43] (*kib*^*1*^/+) (Rh5+: 34.7±2.3%, Rh6+: 65.3±2.3%), or the *sav* deficiency mutation *Df(3R)BSC803* (referred to as *sav*^*df*^ hereafter) (*sav*^*df*^/+) (Rh5+: 33.0±3.7%, Rh6+: 67.0±3.7%) did not show any statistical difference in the ratios of pR8 and yR8 (**Figs 1H and S1A–S1D**), while the heterozygous *mer*^*3*^ flies [48] (*mer*^*3*^/+) had a slight increase in pR8s at the expense of yR8s (Rh5+: 43.7±1.1%, Rh6+: 56.3±1.7%) (**Figs 1H and S**1**E)**. We then analyzed pR8 and yR8 subtypes in the double heterozygous flies for these mutations. The ratios of pR8s had minor increases with corresponding decreases of yR8s in the double heterozygous flies for *wts*^*Zn*^*/kib*^*1*^ (Rh5+: 41.7±6.0%, Rh6+: 58.3±6.0%), *kib*^*1*^*/sav*^*df*^ (Rh5+: 41.5±3.6%, Rh6+: 58.5±3.6%) and *wts*^*Zn*^*/sav*^*df*^ (Rh5+: 40.1±4.1%, Rh6+: 59.9±4.1%) (**Figs 1E and 1H and S1G and S1H**). There was a higher increase in pR8s at the expense of yR8s in *wts*^*Zn*^*/mer*^*3*^ heterozygous retinas (Rh5+: 56.2±1.9%, Rh6+: 43.8±1.9%) and in *mer*^*3*^*/kib*^*1*^ heterozygous retinas (Rh5+: 65.7±9.9%, Rh6+: 34.3±9.9%) (**Figs 1F and 1H and S1F**) compared to heterozygous *mer*^*3*^ (*mer*^*3*^/+) mutants and other double heterozygous flies (**Figs 1H and S1E**). These results showed the heterozygous mutations of the genes in the Hippo pathway can enhance each other’s R8 subtype phenotype, but the effects are too subtle for large-scale screening.

We therefore generated a *wts*^*Zn*^-*kib*^*1*^ chromosome by recombining *wts*^*Zn*^ and *kib*^*1*^ mutations and designed a triple heterozygote-based phenotype enhancement assay. We analyzed pR8 and yR8 subtypes in *wts*^*Zn*^-*kib*^*1*^/*mer*^*3*^ and *wts*^*Zn*^-*kib*^*1*^/*sav*^*df*^ triple heterozygous flies, respectively. The number of pR8s was significantly increased in all *wts*^*Zn*^-*kib*^*1*^/*mer*^*3*^ (Rh5+: 89.0±3.7%) and *wts*^*Zn*^-*kib*^*1*^/*sav*^*df*^ (Rh5+: 90.3±2.1%) flies. In contrast, yR8s were dramatically decreased in these flies (Rh6+: 11.0±3.7% and 9.7±2.1%, respectively) (**Figs 1G and 1H and S1I**). These results indicated that the *wts*^*Zn*^-*kib*^*1*^ heterozygous flies provide a sensitive genetic background, into which introducing one copy of a loss-of-function mutation in a gene of the Hippo pathway to generate triple heterozygous flies can dramatically affect R8 subtypes. Therefore, the triple heterozygote-based assay can be used as an efficient and sensitive tool to screen the *Drosophila* chromosomal deficiencies to identify new regulators of the Hippo pathway.

### Identification of *pyd* as a new PR subtype fate determinant

**Fig 2:**
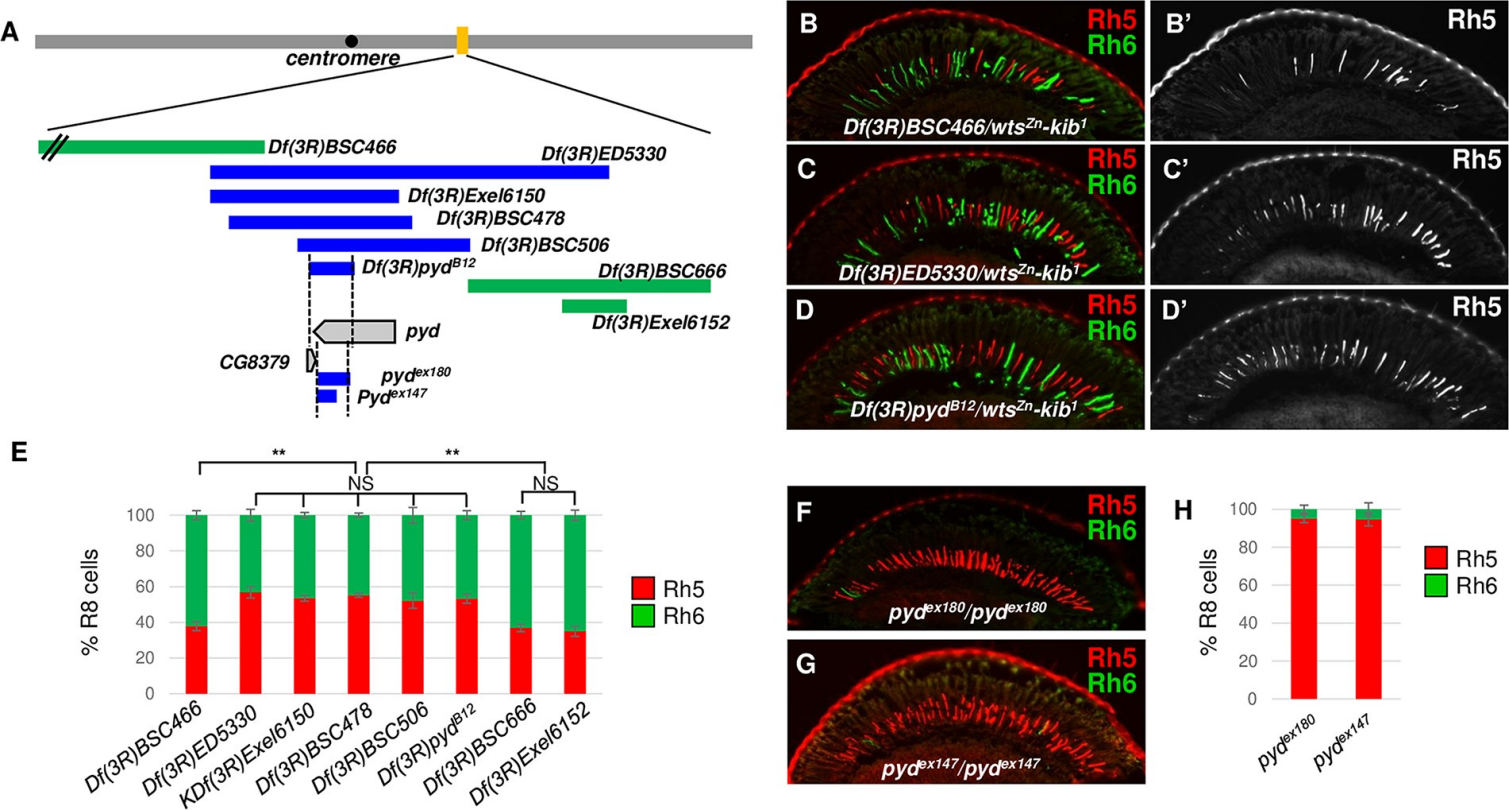
*pyd* was identified to promote yR8 and repress pR8 subtype fate specification. (A) Schematic showing the loci of the deficiencies, the *pyd* mutations and the *pyd* gene. The top grey-shaded bar represents the third chromosome. The yellow bar indicates the relative genomic location of the deletions of the bottom deficiencies and mutations. The green bars indicate the deleted regions of the deficiencies that don’t enhance *wts*^Z*n*^-*kib*^*1*^ phenotype. The blue bars indicate the deleted regions of the deficiencies or mutations that enhance *wts*^*Zn*^-*kib*^*1*^ phenotype. The grey-shaded arrow boxes show the loci of the *pyd* and the *CG8379* genes. (B-D) Adult eye cryosections immunostained for Rh5 (red) and Rh6 (green) from the triple heterozygous flies. (E) Quantification of Rh5- and Rh6-expressing R8s in *wts*^*Zn*^-*kib*^*1*^/deficiency triple heterozygous flies, showing the abilities of each deficiency to enhance *wts*^*Zn*^-*kib*^*1*^ R8 subtype phenotype. y axis presents proportion of R8s that express Rh5 (red) and Rh6 (green). NS: not significant. **P < 0.001. Error bars represent standard deviation. *Df(3R)BSC466*: n = 6 retinas, n = 1145 R8s; *Df(3R)ED5330*: n = 6 retinas, n = 1089 R8s; *Df(3R)Exel6150*: n = 6 retinas, n = 1223 R8s; *Df(3R)BSC478*: n = 6 retinas, n = 1034 R8s; *Df(3R)BSC506*: n = 6 retinas, n = 1002 R8s; *Df(3R)pyd*^*B12*^: n = 6 retinas, n = 1231 R8s; *Df(3R)BSC666*: n = 6 retinas, n = 1100 R8s; *Df(3R)Exel6152*: n = 6 retinas, n = 1090 R8s. (F-G) Adult eye cryosections immunostained for Rh5 (red) and Rh6 (green) in *pyd*^*ex180*^ (F) and *pyd*^*ex147*^ (G) flies. (H) Quantification of Rh5- and Rh6-expressing R8s in the *pyd* mutant flies. *pyd*^*ex180*^: n = 6 retinas, n = 1260 R8s; *pyd*^*ex147*^: n = 6 retinas, n = 1211 R8s.

**Fig 3:**
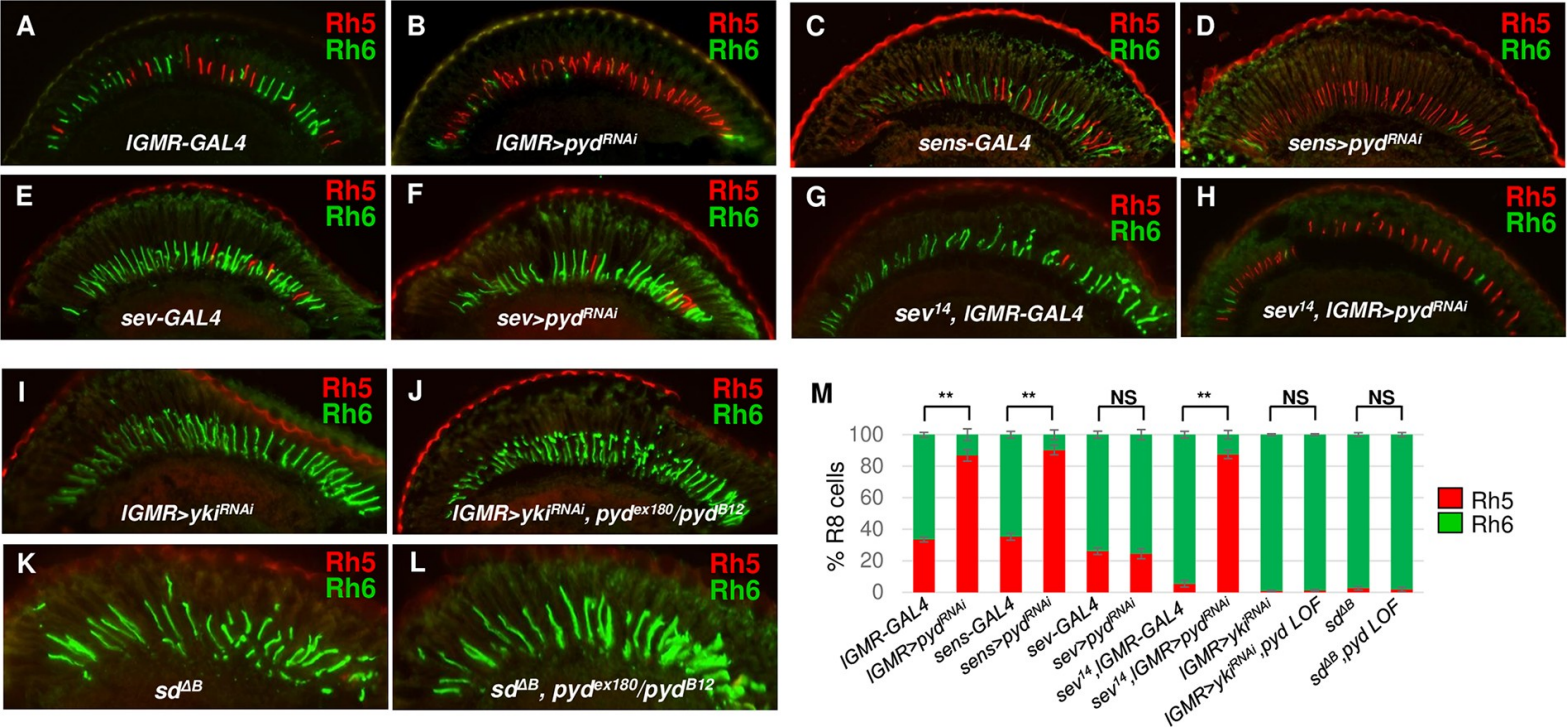
Pyd cell-autonomously specifies R8 subtypes. (A-F) Cryosections of adult eyes stained for Rh5 (red) and Rh6 (green) in control (A, C and E) and *pyd* knock-down flies. Pyd is knocked down in all PRs (B, *lGMR>pyd*^*RNAi*-*KK105581*^), in only R8s (D, *sens>pyd*^*RNAi-kk105581*^) or in R7s (F, *sev>pyd*^*RNAi-KK105581*^). (G-H) Cryosections of *sev* mutant eyes (*sev*^*14*^; *lGMR-GAL4*) (G) and *sev; pyd* double LOF eyes (H, *sev*^*14*^, *lGMR>pyd*^*RNAi*-*KK105581*^). (I-J) Cryosections of the *yki* knock-down (*lGMR>yki*^*RNAi*-*KK109756*^) adult eyes in control (I) or in *pyd* loss of function (LOF*)* (J, *lGMR>yki*^*RNAi*-*KK109756*^, *pyd*^*ex180*^*/pyd*^*B12*^) background. (K-L) Cryosections of *sd* mutant eyes (*sd*^*ΔB*^) (K) and *sd; pyd* double LOF eyes (L, *sd*^*ΔB*^, *pyd*^*ex180*^*/pyd*^*B12*^). (M) Quantification of pR8s (Rh5+) and yR8s (Rh6+) in flies with the indicated genotypes. y axis presents proportion of R8s that express Rh5 (red) or Rh6 (green). NS: not significant. **P < 0.001. Error bars represent standard deviation. *lGMR-GAL4*: n = 10 retinas, n = 1822 R8s; *lGMR>pyd*^*RNAi*^: n = 6 retinas, n = 1093 R8s; *sens-GAL4*: n = 6 retinas, n = 1088 R8s; *sens>pyd*^*RNAi*^: n = 6 retinas, n = 1130 R8s; *sev-GAL4*: n = 6 retinas, n = 1081 R8s; *sev>pyd*^*RNAi*^: n = 6 retinas, n = 1184 R8s; *sev*^*14*^, *lGMR-GAL4*: n = 4 retinas, n = 1006 R8s; *sev*^*14*^, *lGMR>pyd*^*RNAi*^: n = 4 retinas, n = 1027 R8s; *lGMR>yki*^*RNAi*^: n = 4 retinas, n = 805 R8s; *lGMR>yki*^*RNAi*^, *pyd*^*ex180*^*/pyd*^*B12*^: n = 4 retinas, n = 821 R8s; *sd*^*ΔB*^: n = 4 retinas, n = 908 R8s; *sd*^*ΔB*^, *pyd*^*ex180*^*/pyd*^*B12*^: n = 4 retinas, n = 896 R8s.

Taking advantage of the *wts*^*Zn*^-*kib*^*1*^-based triple heterozygote assay, we screened 112 deficiency lines from the Bloomington deficiency Kit, aiming to identify new regulators of the Hippo pathway. The deletions of these 112 deficiencies cover the 61B1 to 88C9 region on the third chromosome (**S2A and S2B Fig**) [55,56]. When crossed with the *wts*^*Zn*^-*kib*^*1*^ chromosome, most of these deficiencies didn’t significantly affect R8 subtype specification (the range of pR8 ratio is 32%-45%, and yR8 ratio is 55%-68%). However, we found that the deficiency *Df(3R)ED5330* significantly increased pR8 ratio at the expense of yR8s (Rh5+: 56.9±3.2%, Rh6+: 43.1±3.2%), compared to the adjacent deficiencies (Rh5+ range: 35.0%-37.9%, Rh6+: 65.0%-62.1%) (**Fig 2A–2C and 2E**). Further analyses of other deficiencies with smaller deletions in this area led us to the deficiency *Df(3R)pyd*^*B12*^ (referred to as *pyd*^*B12*^) [57] (**Fig 2A, 2D and 2E)**, in which partial sequences of two genes, *pyd* and *CG8379*, are deleted (**Fig 2A**). All deficiencies with the deletions of these sequences enhanced the *wts*^*Zn*^-*kib*^*1*^ phenotype to promote pR8 and repress yR8 subtype fate specification (Rh5+ range: 52.2%-55.2%, Rh6+: 47.8%-44.8%) (**Fig 2E**), suggesting either *pyd* or *CG8379* is a PR subtype fate determinant. While there is no mutant allele or RNA interference (RNAi) reagent available for *CG8379*, we analyzed two *pyd* null mutations, *pyd*^*ex180*^ and *pyd*^*ex147*^ [57,58]. pR8 cells were significantly expanded and yR8 cells were significantly reduced in both *pyd*^*ex180*^ (Rh5+: 95.1±2.2%, Rh6+: 4.9±2.2%) and *pyd*^*ex147*^ (Rh5+: 94.6±3.4%, Rh6+: 5.4±3.4%) mutant flies (**Fig 2F–2H**). Additionally, the homozygous *pyd*^*B12*^ flies (*pyd*^*B12*^*/pyd*^*B12*^) or the heteroallelic flies for *pyd*^*B12*^ and pyd^*ex180*^ or another *pyd* mutation *pyd*^*J4*^ [59] demonstrated a similar phenotype (**S3B, S3C, S3F and S3H–S3J Fig)**. Furthermore, knock-down of *pyd* in all photoreceptor cells using *lGMR-GAL4* to drive the expression of independent *pyd* RNAi constructs phenocopied the null *pyd* mutations (**Figs 3A, 3B, S3D, and S3E and S3G**). Together, these results demonstrate that Pyd is a PR subtype fate determinant in the *Drosophila* eye and is required to promote yR8 and repress pR8 subtype fate specification.

### Pyd cell-autonomously regulates the Hippo pathway for R8 subtype fate specification

Because the R8 subtype fate depends on both R7- and R8-dependent events [11,15], we next tested whether Pyd regulates pR8 and yR8 ratios in a cell-autonomous manner. For this purpose, we first used the *sevenless-GAL4* (*sev-GLA4*) and the *senseless-GAL4* (*sens-GAL4*) drivers to knock down *pyd* in R7 and R8 cells, respectively. The two drivers have been previously used to drive the expression of UAS-RNAi constructs and have significantly reduced the expression of their target genes in R7 (*sev-GAL4*) or R8 cells (*sens-GAL4*) [10]. We found knock-down of *pyd* in R8 cells (*sens-GAL4>pyd*^*RANi*^) significantly increased pR8 ratio (Rh5+: 90.2±3.0% vs. 33.5±1.5% in control) at the expense of yR8s (Rh6+: 9.8±0.3% vs. 66.5±1.5% in control) (**Fig 3C, 3D and 3M**). In contrast, knock-down of *pyd* in R7 cells did not affect pR8 and yR8 subtype fates (**Fig 3E, 3F and 3M**). These data suggest Pyd functions cell-autonomously to regulate R8 subtype fate specification. We further confirmed this by knocking down *pyd* in *sevenless* (*sev*) mutant flies. The R7 cell is missing in *sev* mutant flies, and most R8s express Rh6 [13] (Rh5+: 3.5±1.2%, Rh6+: 96.5±1.2% in *sev*^*14*^ flies) (**Fig 3G and 3M**). Knock-down of *pyd* in *sev* mutant flies caused the similar phenotype (Rh5+:87.6±2.2% Rh6+: 12.4±2.2%) (**Fig 3H and 3M**) with *pyd* knock-down in wild type flies (**Fig 3B**). All these results confirm that Pyd functions in R8 cells to specify pR8 and yR8 subtypes.

The phenotype enhancement between *pyd* and *wts*^*Zn*^-*kib*^*1*^ mutations suggests *pyd* is a regulator of the Hippo signaling pathway. To confirm that *pyd* regulates R8 subtype fate specification via regulating Hippo signaling, we analyzed R8 subtypes in *yki* knock-down retinas in *pyd* loss-of-function (LOF) background. Yki is the downstream effector of the Hippo pathway and is required for pR8 subtype fate specification: *yki* knock-down resulted in a dramatic loss of pR8s and an expansion of yR8s (Rh5+: 0.9±0.5%, Rh6+: 99.1±0.5%) [10] (**Fig 3I and 3M)**. We found knock-down of *yki* in *pyd* LOF retinas suppressed *pyd* LOF phenotype and led to a loss of pR8 cells (1.2±0.6%) and an expansion of yR8s (98.8±0.6%) (**Fig 3J and 3M**), suggesting *yki* is downstream of *pyd* during R8 subtype fate decisions. The transcription factor Sd is required to recruit Yki to DNA and regulate its target genes expression during R8 subtype fate specification [10]. We found that *sd* mutation or knock-down was able to suppress *pyd* LOF phenotype (Rh5+: 2.6±1.2%, Rh6+: 97.4±1.2% in *sd* mutant (*sd*^*ΔB*^) eyes; Rh5+: 1.9±1.4%, Rh6+: 98.1±1.4% in *sd*^*ΔB*^, *pyd* LOF eyes) (**Figs 3K–3M and S4A and S4B**), similar with knock-down of *yki*. Altogether, these results demonstrate Pyd functions autonomously in R8s and is required for Hippo signaling to promote yR8 and repress pR8 subtype fate specification.

### Pyd regulates *wts* and *melt* expression in R8 cells

**Fig 4:**
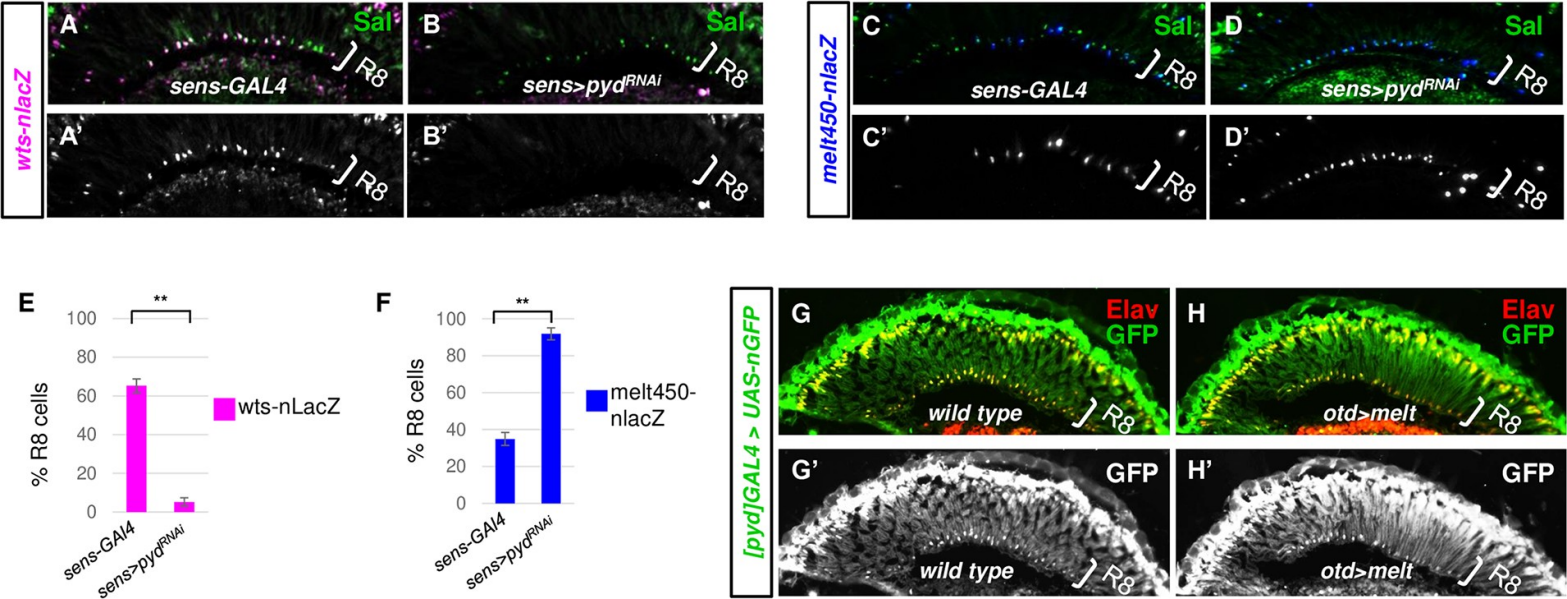
*pyd* regulates the expression of *wts and melt*, while *pyd* expression is not regulated by *wts* or *melt*. (A-B) Cryosections of adult eyes immunostained for LacZ (magenta) and the R8/R7 marker Spalt (Sal, green) showing the expression of *wts* in control (A) and *pyd* knock-down (B, *sens>pyd*^*RNAi-KK105581*^) eyes. Sal labels all R8 cells. (C-D) Cryosections of adult eyes immunostained for LacZ (blue) and the R8/R7 marker Sal (green) showing the expression of *melt450* in control (L) and *pyd* knock-down (M, *sens>pyd*^*RNAi-KK105581*^) eyes. (E-F) Quantification of *wts*-expressing (E) or *melt450*-expressing (F) R8s in control (*sens-GAL4*) and *pyd* knock-down R8 cells (*sens-GAL4>pyd*^*RNAi-KK105581*^). y axis presents proportion of R8s that express LacZ. **P < 0.001. Error bars represent standard deviation. (E) *sens-GAL4*: n = 6 retinas, n = 956 R8s; *sens>pyd*^*RNAi*^: n = 6 retinas, n = 905 R8s. (F) *sens-GAL4*: n = 6 retinas, n = 820 R8s; *sens>pyd*^*RNAi*^: n = 6 retinas, n = 803 R8s. (G-H) Cryosections of adult eyes immunostained for GFP (green) and neuronal cell marker Elav (Red) in wild type (G) and *melt* GOF (H, *otd>melt*) flies.

Previous findings have shown that a key step in dictating pR8 (Rh5-positive) vs. yR8 (Rh6-positive) fate is through the transcriptional activation of *melt* and *wts* in pR8s and yR8s, respectively [15]. To determine whether *pyd* functions upstream of the *melt-wts* regulatory loop, we first analyzed *wts* and *melt* expression in *pyd* knock-down retinas by using an enhancer trap line for *wts* (*wts-nLacZ*, aka *wts*^*Zn*^) [15] and an expression reporter for *melt* (*melt450-nLacZ*) [10]. *wts*^*Zn*^ was expressed in ~65% of R8s (yR8s, 65.2±3.6% of R8s) in wild type retinas (**Fig 4A and 4E**). However, its expression was lost in most of R8s in *pyd* knock-down retinas (5.2±2.1% of R8s) (**Fig 4B and 4E**). In contrast, *melt* expression was expanded into most R8s in *pyd* knock-down retinas (91.9±3.2% of R8s) (**Fig 4D and 4F**), compared to its expression in ~ 35% of R8s in wild type retinas (34.9±3.5%) (**Fig 4C and 4F**). Therefore, these results indicate Pyd is required for *wts* expression and *melt* repression in yR8 cells.

As *pyd* plays a similar role with *wts* to repress *melt* expression, we tested whether *pyd* is also expressed in an yR8-specific manner. We performed a Gal4 enhancer trapping by using the pGawB transposons which insertions are within or adjacent to the *pyd* gene. From the four enhancer trap lines (*p[GawB]*^*NP4419*^, *p[GawB]*^*NP7518*^, *p[GawB]*^*NP0961*^ and *p[GawB]*^*NP4414*^), we found that only the *p[GawB]*^*NP4419*^, which insertion is 8bp upstream of the *pyd* gene, was able to drive the expression of the reporter gene *UAS-nuclear GFP* (*UAS-nGFP*) in PR cells. The expression was found in all PR cells and also in non-neuronal cells (**Fig 4G**). We further tested its expression in *melt* GOF (*otd>melt*) or *wts* GOF (*otd>wts*) flies and found that its expression was not affected by *melt* or *wts* misexpression (**Fig 4H**). All these data suggest that *pyd* is transcriptionally expressed in both pR8 and yR8 subtypes, and its expression is not regulated by *wts* or *melt*.

### Pyd functions upstream of the core components of the Hippo pathway for PR subtype fate specification

**Fig 5:**
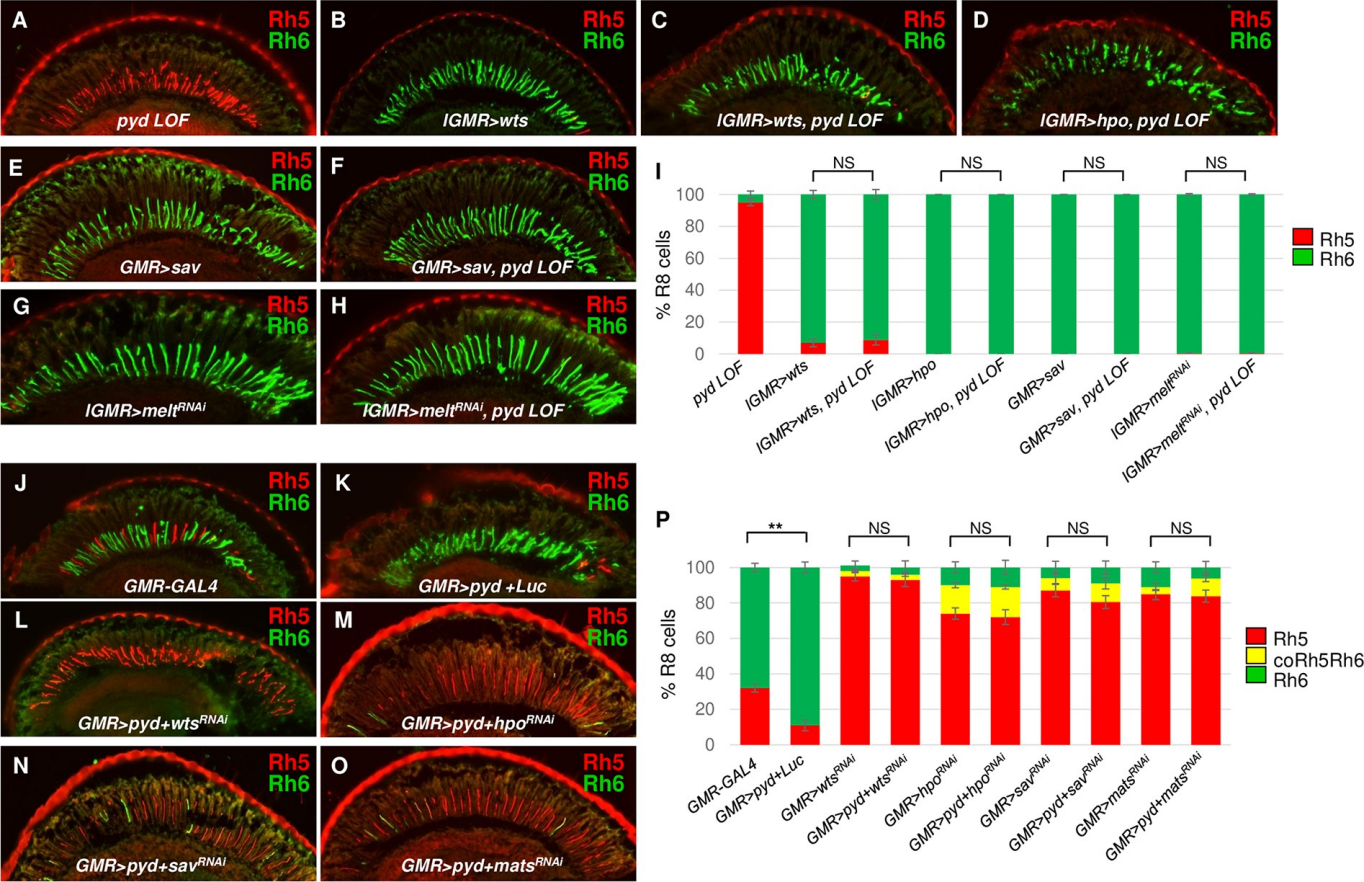
Pyd functions upstream of the core components of the Hippo pathway for R8 subtype fate specification. (A-H) Cryosections of adult eyes in which *wts*, *hpo*, *sav* or *melt* were genetically manipulated as indicated in *pyd* LOF flies were stained for Rh5 (red) and Rh6 (green). LOF = loss-of-function. Genotypes are: (A) *pyd*^*ex180*^*/pyd*^*B12*^, (B) *lGMR>wts*, (C) *lGMR>wts*, *pyd*^*ex180*^*/pyd*^*B12*^, (D) *lGMR>hpo*, *pyd*^*ex180*^*/pyd*^*B12*^, (E) *GMR>sav*, (F) *GMR>sav*, *pyd*^*ex180*^*/pyd*^*B12*^, (G) *lGMR>melt*^*RNAi*^, (H) *lGMR>melt*^*RNAi*^, *pyd*^*ex180*^*/pyd*^*B12*^. Refer to Supporting Information S1 Text for detail genotypes. (I) Quantification of Rh5- and Rh6-expressing R8s in the eyes with the indicated genotypes. y axis presents proportion of R8s that express Rh5 (red) or Rh6 (green). Error bars represent standard deviation. NS: not significant. *pyd* LOF: n = 10 retinas, n = 1902 R8s; *lGMR>wts*: n = 6 retinas, n = 1005 R8s; *lGMR>wts*, *pyd LOF*: n = 6 retinas, n = 1033 R8s; *lGMR>hpo*: n = 4 retinas, n = 823 R8s; *lGMR>hpo*, *pyd* LOF: n = 4 retinas, n = 801 R8s; *GMR>sav*: n = 4 retinas, n = 922 R8s; *GMR>sav*, *pyd* LOF: n = 4 retinas, n = 889 R8s; *lGMR>melt*^*RNAi*^: n = 4 retinas, n = 1134 R8s; *lGMR>melt*^*RNAi*^, *pyd* LOF: n = 4 retinas, n = 1201 R8s. (J-O) Cryosections of adult eyes stained for Rh5 (red) and Rh6 (green) in flies with the indicated genotypes. Genotypes are: (J) *GMR-GAL4*, (K) *GMR>pyd+Luc*, (L) *GMR>pyd+wts*^*RNAi*^, (M) *GMR>pyd+hpo*^*RNAi*^, (N) *GMR>pyd+sav*^*RNAi*^, (O) *GMR>pyd+mats*^*RNAi*^. (P) Quantification of Rh5- and Rh6-expressing R8s in the eyes with the indicated genotypes. y axis presents proportion of R8s that express Rh5 (red) or Rh6 (green). Error bars represent standard deviation. NS: not significant. **P < 0.001. *GMR-GAL4*: n = 6 retinas, n = 930 R8s; *GMR>pyd*+*Luc*: n = 6 retinas, n = 912 R8s; *GMR>wts*^*RNAi*^: n = 6 retinas, n = 1026 R8s; *GMR>pyd*+*wts*^*RNAi*^: n = 6 retinas, n = 810 R8s; *GMR>hpo*^*RNAi*^: n = 5 retinas, n = 891 R8s; *GMR>pyd*+*hpo*^*RNAi*^: n = 5 retinas, n = 801 R8s; *GMR>sav*^*RNAi*^: n = 5 retinas, n = 842 R8s; *GMR>pyd*+*sav*^*RNAi*^: n = 5 retinas, n = 821 R8s; *GMR>mats*^*RNAi*^: n = 5 retinas, n = 921 R8s; *GMR>pyd*+*mats*^*RNAi*^: n = 5 retinas, n = 842 R8s. *UAS-Luciferase* (*UAS-Luc*) was used as a control to balance the number of UAS sites. Refer to Supporting Information S1 Text for detail genotypes.

To further understand how Pyd regulates the Hippo pathway to specify PR subtypes, we performed epistasis assays for Pyd and the core Hippo components in pR8 and yR8 subtype fate specification. Misexpression of *wts*, *hpo* or *sav* in wild type retinas was sufficient to induce yR8 fate and repress pR8 fate in most or all R8 cells [15,49] (Rh5+: 7.1±2.5%, Rh6+: 92.9±2.5% in *lGMR>wts* retinas; Rh5+: 0%, Rh6+: 100% in *lGMR>hpo* retinas; Rh5+: 0%, Rh6+: 100% in *GMR>sav* retinas) (**Fig 5B, 5E and 5I**). We found that misexpression of these core genes *wts*, *hpo* or *sav* in *pyd* mutant retinas had the same abilities to promote yR8 and repress pR8 subtype fate specification with their misexpression in wild type retinas: with the misexpression of *wts*, *hpo*, or *sav*, all or most of pR8s in *pyd* mutant retinas adopted to yR8 subtype fate (Rh5+: 8.8±3.1%, Rh6+: 91.2±3.1% in *lGMR>wts*, *pyd LOF* retinas; Rh5+: 0%, Rh6+: 100% in *lGMR>hpo*, *pyd LOF* retinas; Rh5+: 0%, Rh6+: 100% in *GMR>sav*, *pyd LOF* retinas) (**Fig 5C, 5D, 5F and 5I**). To further confirm the epistasis of *pyd* and *wts*, we performed *pyd* and *melt* double LOF experiments. *wts* expression in pR8s is derepressed in *melt* mutant flies [15]. We knocked down *melt* in *pyd* mutant (*pyd*^*ex180*^*/pyd*^*B12*^) flies or knocked down *pyd* in *melt* mutant (*melt*^*Δ1*^) flies. Loss of *melt* in both experiments suppressed the phenotype caused by loss of *pyd* (Rh5+: 0.4±0.6%, Rh6+: 99.5±0.6% in *lGMR>melt*^*RNAi*^ flies; Rh5+: 0.5±0.8%, Rh6+: 99.5±0.8% in *lGMR>melt*^*RNAi*^, *pyd* LOF flies) (**Figs 5G–5I and S5A–S5F**). Together, these results indicate that Pyd genetically acts upstream of or in parallel to the core components of the Hippo pathway.

The expression of *pyd* in both pR8 and yR8 subtypes indicates that the Pyd protein at its endogenous level in pR8 cells might not be sufficient to induce yR8 fate. In order to test whether the core components of the Hippo pathway are necessary for Pyd-mediated yR8 fate specification, we tested whether overexpression of Pyd in all PR cells by using a strong GMR-GAL4 driver [60] (*GMR-GAL4>UAS-pyd*) can induce yR8 fate specification. We found that *pyd* overexpression induced Rh6 expression in most R8 cells (Rh6+: 88.9±3.1%). Rh5 expression was only observed in a small proportion of R8s in *pyd* overexpression retinas (Rh5+: 11.1±3.1%) (**Fig 5K and 5P**). We then knocked down *wts*, *hpo*, *sav* or *mats* in *pyd*-overexpressing retinas, and found that knocking down any of these genes abolished the overexpressed Pyd’s ability to promote yR8 and suppress pR8 fate specification (Rh5+: 93.0±3.7%, Rh6+: 4.1±3.7%, Rh5 and Rh6 co-expression (coRh5Rh6): 2.9±1.1% in *GMR>wts*^*RNAi*^, *pyd GOF* flies; Rh5+: 72.5±4.1%, Rh6+: 10.4±4.1%, coRh5Rh6: 17.1±1.2% in *GMR>hpo*^*RNAi*^, *pyd GOF* flies; Rh5+: 80.5±3.6%, Rh6+: 9.0±3.6%, coRh5Rh6: 10.5±3.1% in *GMR>sav*^*RNAi*^, *pyd GOF* flies; Rh5+: 83.5±3.4%, Rh6+: 6.2±3.4%, coRh5Rh6: 10.3±1.8% in *GMR>mats*^*RNAi*^, *pyd GOF* flies) (**Fig 5L–5P**), suggesting these Hippo core components are necessary for Pyd to promote yR8 and inhibit pR8 subtype fate specification. Together, these epistasis experiments suggested Pyd genetically functions upstream of the core component genes in the Hippo pathway for R8 subtype fate decisions.

### Pyd functions upstream of *pez* for R8 subtype fate specification

**Fig 6:**
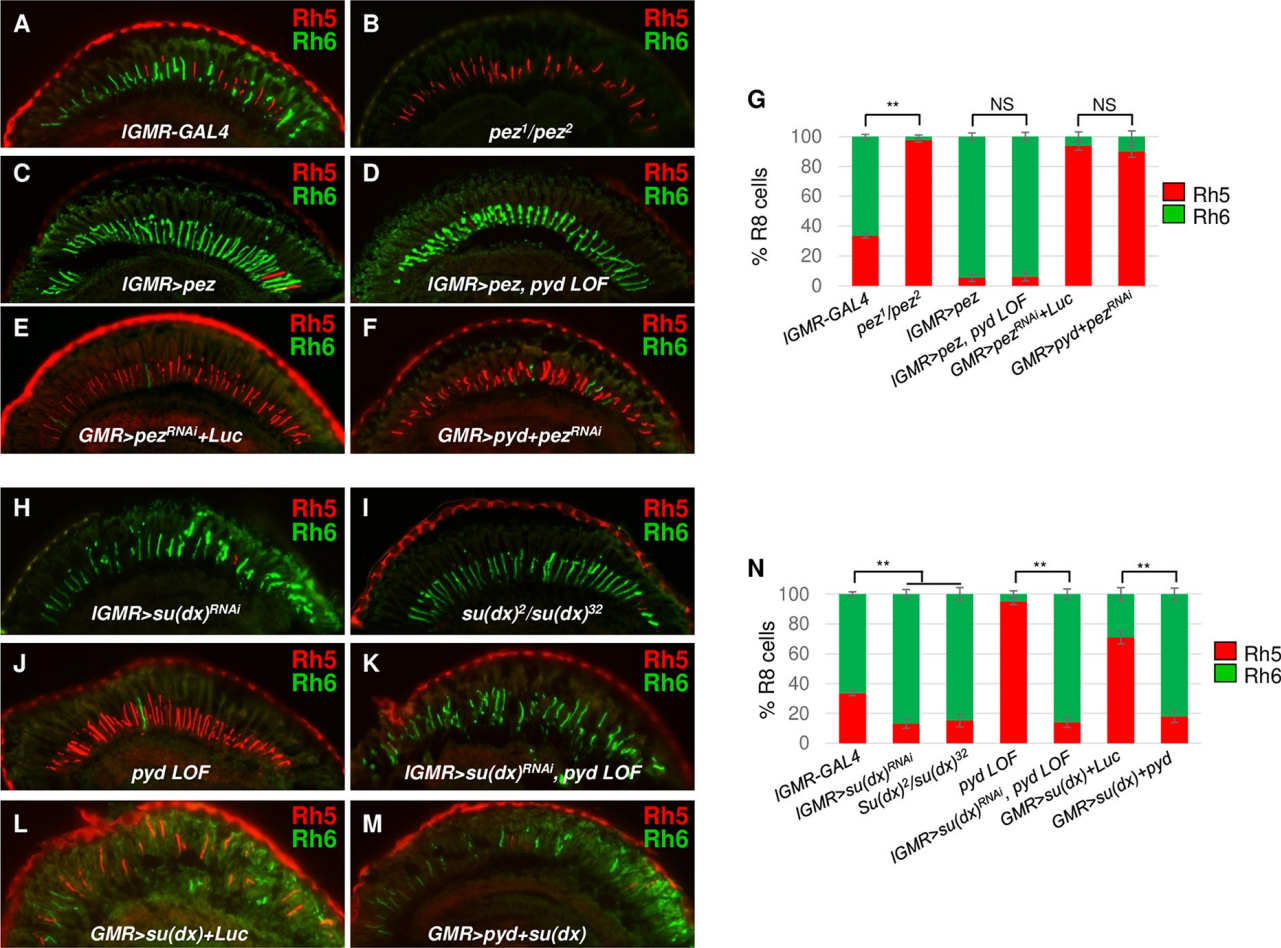
*pez* and *su(dx)* specify R8 subtypes downstream of *pyd*. (A-F) Cryosections of adult eyes stained for Rh5 (red) and Rh6 (green) in control (*lGMR-GAL4*) (A), *pez* mutant (*pez*^*1*^*/pez*^*2*^) (B), *pez* misexpression (*lGMR>pez*) (C), *pez* misexpression in *pyd* LOF (*lGMR>pez*, *pyd*^*ex180*^*/pyd*^*B12*^) (D), knock-down of *pez* (*GMR>pez*^*RNAi*^*+Luc*) (E) and knock-down of *pez* in *pyd* overexpression (*GMR>pyd+pez*^*RNAi*^) (F) flies. (G) Quantification of Rh5 and Rh6-expressing R8s in the eyes with the indicated genotypes. y axis presents proportion of R8s that express Rh5 (red) or Rh6 (green). NS: not significant. **P < 0.001. Error bars represent standard deviation. *lGMR-GAL4*: n = 10 retinas, n = 1822 R8s; *pez*^*1*^*/pez*^*2*^: n = 6 retinas, n = 967 R8s; *lGMR>pez*: n = 6 retinas, n = 1201 R8s; *lGMR>pez*, *pyd* LOF: n = 6 retinas, n = 1261 R8s; *GMR>pez*^*RNAi-KK*^*+Luc*: n = 6 retinas, n = 1001 R8s; *GMR>pyd+pez*^*RNAi*^: n = 5 retinas, n = 936 R8s. (H-M) Cryosections of adult eyes stained for Rh5 (red) and Rh6 (green) in *su(dx)* knock-down (H, *lGMR>su(dx)*^*RNAi*^), *su(dx)* mutant (I, *su(dx)*^*2*^*/su(dx)*^*32*^), *pyd* LOF (*pyd*^*ex180*^*/pyd*^*B12*^) (J), *su(dx)* knock-down in *pyd* LOF (*lGMR> su(dx)*^*RNAi*^, *pyd*^*ex180*^*/pyd*^*B12*^) (K). *su(dx)* GOF (*GMR>su(dx)+Luc*) (L) and *su(dx)*, *pyd* double GOF (*GMR>su(dx)+pyd*) (M) flies. (N) Quantification of Rh5- and Rh6-expressing R8s in the eyes with the indicated genotypes. y axis presents proportion of R8s that express Rh5 (red) or Rh6 (green). **P < 0.001. Error bars represent standard deviation. *lGMR-GAL4*: n = 10 retinas, n = 1822 R8s; *lGMR>su(dx)*^*RNAi*^: n = 6 retinas, n = 903 R8s; *su(dx)*^*2*^*/su(dx)*^*32*^: n = 6 retinas, n = 1038 R8s; *pyd* LOF: n = 10 retinas, n = 1902 R8s; *lGMR>su(dx)*^*RNAi*^, *pyd* LOF: n = 6 retinas, n = 980 R8s; *GMR*>*su(dx)+Luc*: n = 4 retinas, n = 823 R8s; *GMR*>*su(dx)+pyd*: n = 4 retinas, n = 802 R8s.

Pyd was previously shown to directly interact with the E3 ubiquitin ligase Su(dx) in regulating the size of the *Drosophila* ovary stem cell niche [58]. Additionally, Su (dx) targets and degrades Pez during intestinal stem cell proliferation [52]. Pez is the *Drosophila* homolog of Protein tyrosine phosphatase non-receptor type 14 (PTPN14) and functions as a partner of Kib. Both Pez and Kib are required for the activity of the Hippo pathway to restrict intestinal stem cell proliferation in the *Drosophila* midgut epithelium [51]. However, the roles of both Pez and Su(dx) in post-mitotic PR subtype fate specification have not been explored. Here, we first analyzed pR8 and yR8 subtypes in heteroallelic *pez*^*1*^/*pez*^*2*^ mutant flies. Almost all R8s expressed Rh5 (97.5±1.2%), the pR8 fate marker, in *pez*^*1*^/*pez*^*2*^ mutant flies at the expense of Rh6 (2.5±1.2%) (**Fig 6B and 6G**). Furthermore, misexpression of *pez* (*lGMR*>*pez*) led to a significant increase in Rh6-expressing yR8s (94.6±2.5%) and a reduction in Rh5-expressing pR8s (5.4±2.5%) (**Fig 6C and 6G**). These results demonstrate that *pez* is necessary and sufficient to promote yR8 and repress pR8 subtype fate specification in the *Drosophila* eye. We then determined the genetic relationship between *pyd* and *pez*. Misexpression of *pez* was able to suppress the phenotype caused by *pyd* mutations (Rh5+: 6.1±2.9%, Rh6+: 93.9±2.9% in *lGMR>pez*, *pyd LOF* retinas) (**Fig 6D and 6G**), similar with misexpression of the core components of the Hippo pathway. Further, we knocked down *pez* in *pyd*-overexpressing eyes and found that loss of *pez* repressed the phenotype caused by overexpressed Pyd (Rh5+: 90.2±3.9%, Rh6+: 9.8±3.9% in *GMR>pez*^*RNAi*^+*pyd* retinas) (**Fig 6E–6G**), indicating Pez is required for Pyd to promote yR8 and repress pR8 subtype fate. Together, these results indicated that Pez acts downstream of Pyd in the Hippo pathway to specify R8 subtype fate specification.

### Pyd suppresses Su(dx) in R8 subtype fate specification

Su(dx) plays an opposite role to Pyd in regulating the size of the ovary stem cell niche [58]. In order to investigate the functional relationship of the two proteins in R8 subtype fate specification, we first tested whether Su(dx) plays any role in PR subtype fate specification by knocking down *su(dx)* in retinas. Knock-down of *su(dx)* (*lGMR*>*su(dx)*^*RNAi*^) led to an opposite phenotype to loss of *pez*: Rh6-expressing yR8s were significantly increased (86.8±3.1%) at the expense of Rh5-expressing pR8s (13.2±3.1%) (**Fig 6H and 6N**). We additionally used *su(dx)* mutations *su(dx)*^*2*^ and *su(dx)*^*32*^ to generate heteroallelic *su(dx)*^*2*^/*su(dx)*^*32*^ mutant flies and analyzed R8 subtypes in these flies. *su(dx)*^*2*^/*su(dx)*^*32*^ retinas showed a similar R8 subtype phenotype (Rh5+: 15.2±4.3%, Rh6+: 84.8±4.3%) with *su(dx)* knock-down retinas (**Fig 6I and 6N**). Further, we found that misexpression of *su(dx)* (*lGMR>su(dx*)) caused an increase in pR8s (71.1±4.8%) and a reduction in yR8s (28.9± 4.8%) (**S6A–S6C Fig**). Therefore, Su(dx) and Pyd play opposite roles in R8 subtype fate decisions.

Previous yeast two-hybrid assays and co-immunoprecipitation (co-IP) tests in the *Drosophila* S2 cells have shown that Pyd and Su(dx) can interact with each other and form a complex. To explore the functional relationship of the two interacting proteins during R8 subtype fate specification, we performed epistasis analyses for *pyd* and *su(dx)*. We knocked down *su(dx)* in *pyd* mutant flies and found that knock-down of *su(dx)* suppressed the increase of the number of pR8s and the decrease of yR8s caused by *pyd* mutations (Rh5+: 14.1±3.5%, Rh6+: 85.9±3.5% in *lGMR*>*su(dx)*^*RNAi*^, *pyd LOF* retinas; compared to Rh5+: 95.1±2.2%, Rh6+: 4.9±2.2% in *pyd LOF* retinas) (**Fig 6J, 6K and 6N**). These results suggest that Pyd genetically functions upstream of Su(dx) and that the pR8 expansion phenotype in *pyd* mutant flies depends on the presence of functional Su(dx). We then looked into the effect of Pyd on the misexpressed Su(dx). As shown above, misexpression of Su(dx) leads to an expansion of pR8s at the expense of yR8s. We found that overexpression of Pyd suppressed the ability of Su(dx) to increase pR8s and decrease yR8s (Rh5+:18.7±4.0%, Rh6+: 81.3±4.0% retinas in *GMR>pyd+su(dx)* retinas; compared to Rh5+: 71.1±4.3%, Rh6+: 28.9±4.3% in *GMR*>*su(dx)* retinas) (**Fig 6L–6N**). Considering that Pyd and Su(dx) can physically interact with each other and form a complex, these double LOF and double GOF results indicate Pyd acts through suppressing Su(dx) to regulate R8 subtype fate specification.

### Pyd functions genetically in parallel to Kib to specify R8 subtypes

**Fig 7:**
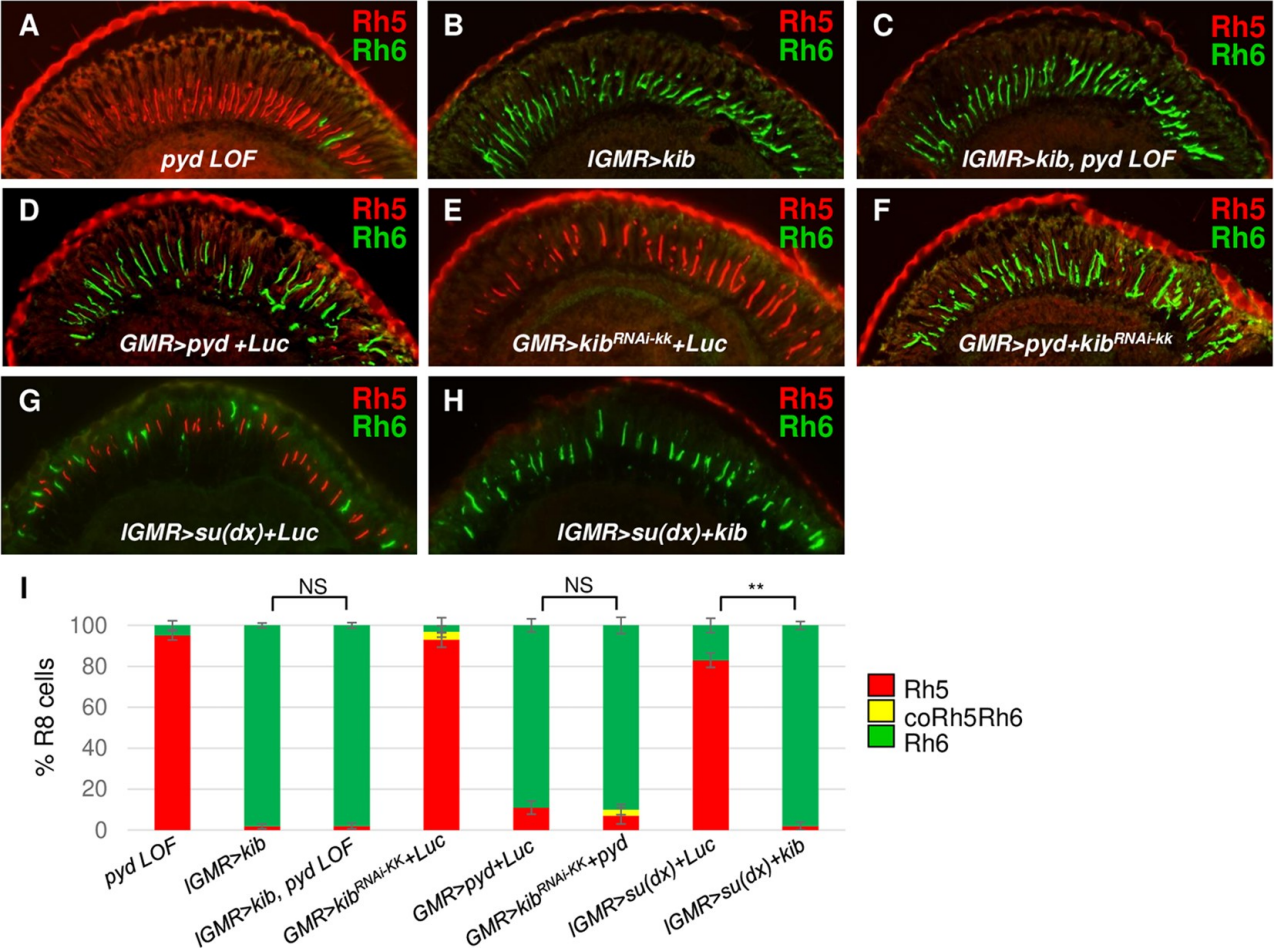
Pyd genetically functions in parallel to *kib* to specify R8 subtypes. (A-H) Cryosections of adult eyes stained for Rh5 (Red) and Rh6 (Green) in *pyd* LOF (A, *pyd*^*ex180*^*/pyd*^*B12*^), *kib* misexpression (B, *GMR>kib*), *kib* misexpression in *pyd* LOF (C, *GMR>kib*, *pyd*^*ex180*^*/pyd*^*B12*^), *pyd* overexpression (D, *GMR>pyd+Luc*), *kib* knock-down (E, *GMR>kib*^*RNAi-kk*^*+Luc*), *kib* knock-down in *pyd* misexpression (F, *GMR>pyd*+*kib*^*RNAi-kk*^), *su(dx)* GOF (G, *lGMR>su(dx)+Luc*) and *su(dx)*, *kib* double GOF (H, *lGMR>su(dx)+Luc*) flies. *UAS-Luciferase* (*UAS-Luc*) was used as a control to balance the number of UAS sites. (I) Quantification of Rh5 and Rh6-expressing R8s in the eyes with the indicated genotypes. NS: not significant. **P < 0.001. Error bars represent standard deviation. *pyd* LOF: n = 10 retinas, n = 1902 R8s; *lGMR>kib*: n = 6 retinas, n = 1020 R8s; *lGMR>kib*, *pyd* LOF: n = 6 retinas, n = 1002 R8s; *GMR>kib*^*RNAi-KK*^*+Luc*: n = 4 retinas, n = 934 R8s; *GMR>pyd*+*Luc*^*RNAi*^: n = 6 retinas, n = 912 R8s; *GMR>pyd+kib*^*RNAi-KK*^: n = 4 retinas, n = 1002 R8s; *lGMR>su(dx)*+*Luc*: n = 4 retinas, n = 830 R8s; *lGMR>su(dx)+kib*: n = 4 retinas, n = 1052 R8s.

Kib can bind to Pez and the two proteins function together to regulate Hippo signaling in the *Drosophila* midgut epithelium [51]. To test whether Kib also genetically functions downstream of Pyd to specify R8 subtypes, we misexpressed *kib* in *pyd* mutant retinas. Similar to *pez*, *kib* misexpression was also able to suppress *pyd* mutant phenotype (Rh5+: 1.8±1.2%, Rh6+: 98.2±1.2% in *lGMR>kib*, *pyd LOF* retinas) (**Fig 7B, 7C and 7I**), suggesting *pyd* functions upstream of or in parallel to Kib in R8 subtype fate specification. We then tested whether Pyd’s ability to induce yR8 subtype requires Kib. We knocked down *kib* in *pyd*-overexpressing retinas (*GMR>pyd+kib*^*RNAi*^) and found that knock-down of *kib* was not able to affect the overexpressed Pyd to promote yR8 and repress yR8 subtype fate (Rh5+: 8.2±4.2%, Rh6+: 88.3±4.2%, coRh5Rh6: 3.5±2.5% in *GMR>kib*^*RNAi*^, *pyd GOF* flies) (**Figs 7D–7F and 7I and S7A–S7D**), although *kib* knock-down in wild type flies is sufficient to repress yR8 fate specification (Rh5+: 92.1±3.7%, Rh6+: 3.4±3.7%, coRh5Rh6: 4.5±2.7% in *GMR>kib*^*RNAi*^ flies (**Figs 7E and 7I, and S7A and S7D**), indicating the overexpressed Pyd is able to circumvent knock-down of *kib* to specify R8 subtypes. Collectively, these results suggest that Pyd and Kib may function genetically in parallel to each other during R8 subtype fate specification.

Kib was previously shown to block the Su(dx)-induced Pez degradation in the cultured *Drosophila* S2 cells [52]. We therefore asked whether Kib is able to suppress Su(dx)’s activity in R8 subtype fate specification. To test this, we misexpressed Kib in Su(dx)-misexpressing retinas. Compared to most R8s expressing Rh5 in Su(dx)-misexpressing retinas (Rh5+: 71.1±4.8%, Rh6+: 28.9± 4.8%) (**Fig 7G and 7I**), *kib* misexpression suppressed Su(dx)’s activity, leading most R8s expressing Rh6 at the expense of Rh5 (Rh5+: 2.1±1.9%, Rh6+: 97.9± 2.1%) (**Fig 7H and 7I**). These results suggested that Kib, similar with Pyd, can suppress Su(dx)’s activity in R8 subtype fate specification.

## Discussion

**Fig 8:**
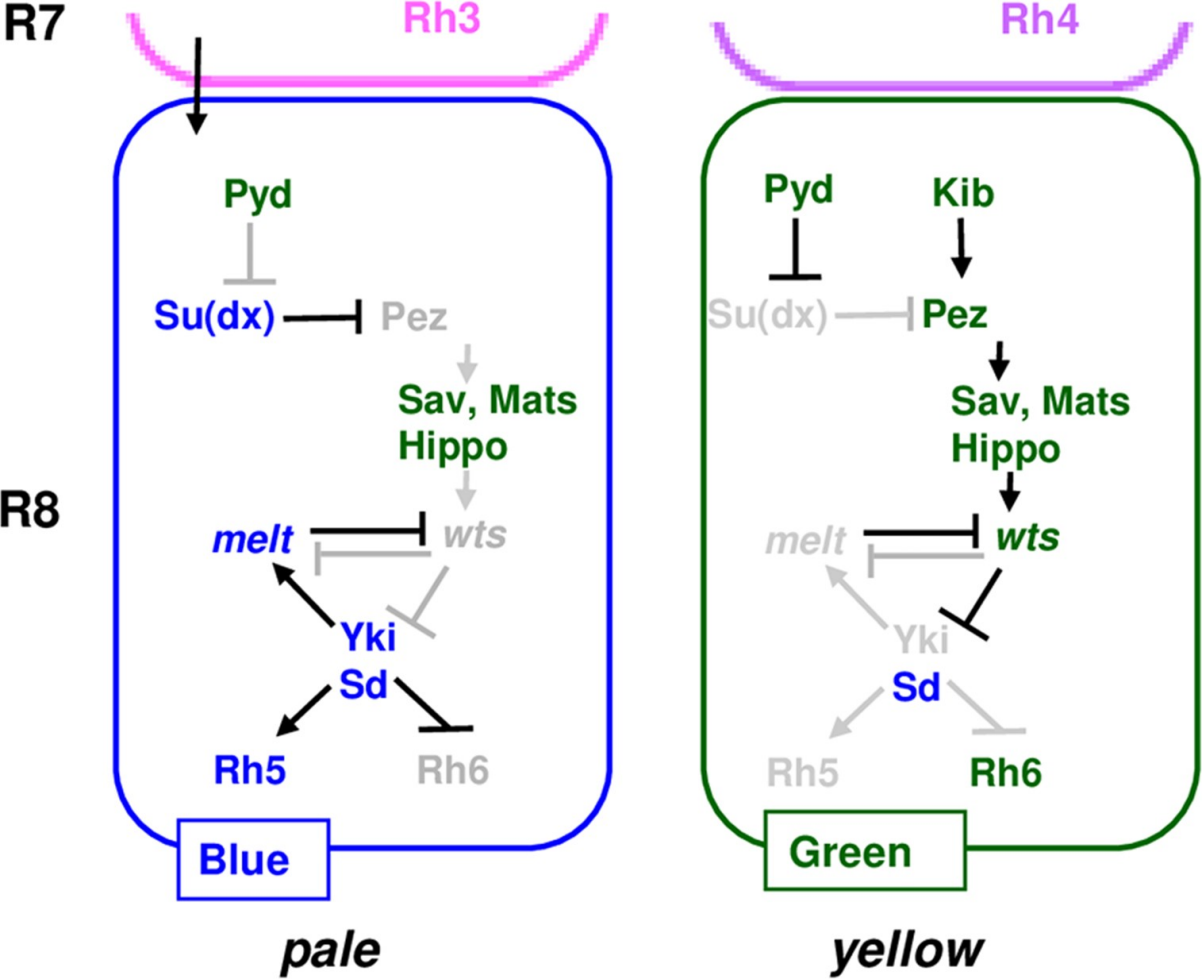
Model: the role of Pyd in R8 subtype fate specification. Pyd represses Su(dx) and may function in parallel to Kib to promote yR8 and repress pR8 subtype fate specification. Pyd may antagonize the Su(dx)-mediated Pez degradation. See Discussion for detail. Genes or proteins that are inactive or not expressed are denoted by grey font, while those that are expressed and/or active are represented by blue, green or black font. Model is based on the data in this study and the previous works [10,15,49,52].

In this study, we designed a sensitized genetic screen using a triple heterozygote-based PR subtype phenotype enhancement assay to identify novel regulators of the Hippo pathway in the *Drosophila* eye. Taking advantage of this genome-wide screening, we identified the *Drosophila* ZO-1 protein Pyd as a new PR subtype fate determinant. We demonstrated Pyd is an upstream regulator of the Hippo signaling pathway and is required for the pathway to promote yR8 and repress pR8 PR subtype fate specification. We also determined the roles of Pez and Su(dx) in R8 subtype fate specification and found they play opposite roles in this process (**Fig 8**), as they act in intestinal stem cell proliferation. Previous reports have shown that Pyd and Su(dx) can physically interact with each other. We found that Pyd and Su(dx) act antagonistically during R8 subtype fate specification (**Fig 8**). Further, our *pyd* and *su(dx)* double LOF and double GOF results have indicated that the R8 subtype phenotype in *pyd* LOF retinas depends on the presence of Su(dx), and on the other hand, the overexpressed Pyd represses Su(dx)’s activity to promote pR8 and inhibit yR8 fate specification. Considering that Su(dx) can induce Pez degradation [52], Pyd may be required for Hippo signaling by antagonizing Su(dx)’s activity and therefore stabilizing Pez (**Fig 8**). Interestingly, it is the WW domain of the Su(dx) protein that interacts with both Pez and Pyd. Therefore, it is possible that Pyd competes with Su(dx) to bind and stabilize Pez. Our data also showed that Kib suppresses Su(dx)’s activity during R8 subtype fate specification, consistent with the previous report that Kib can block Su(dx)-induced Pez degradation [52]. However, Kib was not shown to interact with Su(dx) and it can’t decrease the binding between Su(dx) and Pez [52]. Therefore, Pyd and Kib may use different mechanisms to stabilize Pez: Pyd competes with Su(dx) for Pez binding, while Kib directly binds to Pez. Since loss of *pyd* or *kib* lead to significant expansion of pR8s and reduction of yR8s, both of the two mechanisms is required in wild type retinas. However, overexpression of any one of *pyd* and *kib* can circumvent loss of another gene (**Fig 7**), suggesting the two mechanisms might function independently (**Fig 8**). It will be of interest and important to test this model using biochemical approaches in future studies and explore whether and how Pyd directly competes with Su(dx).

The Hippo pathway was originally discovered in *Drosophila* and its evolutionarily conserved roles in various biological processes have been subsequently found in mammals [19,34,61–68]. However, the regulatory mechanisms upstream of the signaling pathway are less understood. Most of the core components of the Hippo pathway were first isolated as a result of their overgrowth phenotypes in mosaic mutant-based screens [69]. However, this strategy is not efficient to identify the upstream components of the pathway because the overgrowth defects caused by mutations of the upstream genes is much weaker compared to those induced by mutations of the core components due to the redundant roles of the upstream components in tissue growth control [42,43,51,70]. Interestingly, Fat, Expanded as well as Crumbs are not required for the activation of Hippo signaling during R8 subtype fate specification [49], making the upstream regulation of the Hippo pathway during R8 subtype fate decisions less complicated. Additionally, Hippo-dependent R8 subtype fate specification can be precisely quantified. Taking advantage of these features, we generated a sensitive genetic background with double heterozygous *wts* and *kib* mutations that affect R8 subtypes modestly, but is able to significantly change pR8 and yR8 subtypes when coupled with one more mutation in a gene of the Hippo pathway. This sensitive genetic tool makes it possible to perform a genome-wide screening by testing the activities of the *Drosophila* deficiency lines to affect R8 subtypes. Notably, previous studies have demonstrated that Mer physically interacts with Wts and Kib [42,71]. Our results showed the R8 subtype phenotype was enhanced more in heterozygous *wts*^*Zn*^-*mer*^*3*^ or *kib*^*1*^-*mer*^*3*^ flies than in *wts*^*Zn*^*-kib*^*1*^, *kib*^*1*^*-sav*^*df*^ or *wts*^*Zn*^*-sav*^*df*^ flies. Therefore, the quantitative phenotype enhancement assays for R8 subtypes have a potential application to predict the physical interactions between the components of the Hippo pathway.

ZO-1 proteins are crucial for the formation and maintenance of tight junctions in vertebrate cells [72]. While in *Drosophila* cells, which lack tight junctions [73], Pyd is associated with both adherens and septate junctions [74,75]. ZO-1 proteins are members of the membrane-associated guanylate kinase (MAGUK) family and contain a GUK (guanylate kinase) domain, three PDZ domains, and a SH3 domain [58]. The cellular localization and the presence of multiple protein-protein interaction domains suggest the ZO-1 proteins may play important roles in coupling the extracellular signals to intracellular signaling pathways. A previous study in cultured cells have found that the transiently expressed ZO-1 protein can interact with the carboxy-terminal PDZ binding motif of TAZ, a downstream effector of the Hippo pathway in mammals, via its first PDZ domain [76]. Whether Pyd interacts with Yki, the *Drosophila* homolog of YAP/TAZ, hasn’t been explored. However, our result that knock-down of *wts* is sufficient to suppress the phenotype in *pyd* overexpression retinas suggests the interaction between Yki and Pyd, if there is any, does not play a significant role for the cytoplasmic retention of Yki and thus inhibiting its activity as a transcription co-activator. Additionally, Pyd has been previously implicated in the regulation of the Notch pathway in context-dependent manners [59,77,78]. However, the Notch pathway has not been shown to cell-autonomously regulate R8 subtype fate specification in the *Drosophila* eye. Our results in this study provide evidence that Pyd is a regulator of the Hippo signaling pathway and functions as an upstream regulator of the pathway for PR subtype fate decisions. Considering its interactions with junctions and cytoskeleton proteins, Pyd might function as a scaffold to organize other components of the Hippo pathway at the plasma membrane to form functional complexes. Furthermore, genetic or direct interactions between Pyd and some transmembrane proteins have been previously reported [79,80]. Given that Pyd functions upstream of the Hippo pathway during R8 subtype fate decisions, it will be of great interest to test the role of these Pyd-interacting transmembrane proteins for R8 subtype fate decisions and investigate whether any of them acts as a transmembrane receptor in Hippo signaling. Notably, Mer plays key roles to recruit the core Hippo components to apical membrane area [81]. Pyd and its transmembrane partner may be required for Mer membrane associations.

The R8 terminal differentiation into pR8 or yR8 subtype fate occurs in the late pupal stage and is dependent on the activation and deactivation of the Hippo signaling pathway [82]. As a negative regulator of the Hippo pathway, *melt* is expressed in a subset of R8s from 40% pupation [10] and is indispensable to transcriptionally repress *wts* expression and de-activate Hippo signaling [49], allowing these R8s to adopt the pR8 subtype fate. In this study, we determined that the E3 ligase Su(dx) as another negative mediator of the Hippo pathway for R8 subtype fate specification. Su(dx) was shown to degrade Pez and therefore inactivate Hippo signaling in midgut epithelium [52], indicating Su(dx) inactivates Hippo signaling by a different mechanism with Melt-mediated transcriptional repression of *wts*. It is possible that Su(dx) is present in a subset of R8s at 40–50% APF stage and its presence reduces the default Hippo signaling and thus results in elevated Yki activity which, together with the transcription factors Otd, Traffic jam and Scalloped [10,20], initiates the *melt*-*wts* bistable loop to activate *melt* and repress *wts* expression, and finally leads to the generation of pR8 subtype.

## Materials and methods

### Drosophila stocks

The following fly lines were used: *pyd*^*ex180*^, *pyd*^*ex147*^, *UAS-GFP-pyd* [58], *kib*^*1*^, *pez*^*1*^, *pez*^*2*^, *UAS-kib*, *UAS-pez* [43,51], *wts*^*Zn*^ (*wts-nLacZ*) [15,17], *melt*^*Δ1*^ [16], *sd*^*ΔB*^ [83], *lGMR-GAL4* [84], *UAS-pyd*^*RANi-#450*^ [79], *sensR8-GAL4* [11], *yki*^*B5*^ [33], *UAS-hpo* [26], *GMR-sav* [32], *pWIZ-wΔ13* (a *white* gene RNAi line) [85]. *UAS-pyd*^*RANi*^ (KK105581), *UAS-yki*^*RNAi*^ (KK109756), *UAS-sd*^*RNAi*^ (KK108877), *UAS-wts*^*RNAi*^ (KK101055), *UAS-hpo*^*RNAi*^ (KK101704), *UAS-sav*^*RNAi*^ (KK107562), *UAS-mats*^*RNAi*^ (KK100140) and *UAS-kib*^*RNAi*^ (KK108510) were from the Vienna Drosophila Resource Center (VDRC). *UAS*-*pyd*^RNAi^ (HMS00263) *UAS-wts*, *UAS-su(dx)*, *su(dx)*^*2*^, *su(dx)*^*32*^, *sev*^*14*^, *UAS-su(dx)*^*RNAi*^ (HMS05478), *UAS-pez*^*RNAi*^ (HMS00862), *UAS-kib*^*RNAi*^ (HMC03256), *UAS-Dicer2*, *sev-GAL4*, *GMR-GAL4* [60], *UAS-Luciferase*, *Df(3R)BSC803 (sav*^*df*^*)*, *Df(3R)ED6096*, *Df(3R)BSC466*, *Df(3R)ED5330*, *Df(3R)Exel6150*, *Df(3R)BSC478*, *Df(3R)BSC506*, *Df(3R)BSC666*, *Df(3R)Exel6152*, and *Df(3R)pyd*^*B12*^ were from the Bloomington Drosophila Stock Center (BDSC). *p[GawB]*^*NP4419*^, *p[GawB]*^*NP7518*^, *p[GawB]*^*NP0961*^ and *p[GawB]*^*NP4414*^ were from the Kyoto Stock Center. *lGMR-GAL4*, *pWIZ-wΔ13* and *UAS-Dicer2* lines were recombined onto a single chromosome for use in RNAi-mediated knockdown experiments [10]. *UAS-pez* and *pyd*^*ex180*^, both on the 3^rd^ chromosome, were recombined and used for misexpression of *pez* in *pyd LOF* flies. All flies and crosses were raised on standard cornmeal-molasses media at 25°C with 12 hr:12hr light-dark cycles except that *GMR-GAL4>UAS-su(dx)* (for Fig 6L and 6M) were in room temperature (22°C).

### Immunohistochemistry

Fly head cryosections, dissection for whole mount retinas, and antibody staining were performed as previously described with modifications [82,86]. Adult fly heads were embedded and frozen in OCT and sectioned (12 μm) using the Cryostat CM1850 (Leica). The samples were then fixed in 4% paraformaldehyde/ PBS, and washed 3x10 min with PBX (PBS + 0.3% Triton X-100), incubated with primary antibodies overnight at 4°C in antibody dilution buffer (PBX + 1% BSA), washed 4x10 min with PBX, and incubated 90 min at room temperature with secondary antibodies diluted in antibody dilution buffer. After 4x10min PBT washes, samples were mounted in anti-fade reagent, and imaged. Antibody dilutions were: rabbit Salm (1:150) [82]; mouse Rh5 (1:1000) [87]; rabbit Rh6 (1:100, this study); chicken LacZ (1:1000, Abcam). Alexa Fluor 488, 555 and 655-conjugated secondary antibodies (1:1500, Invitrogen) were used. Digital images were obtained with an Apotome deconvolution system (Zeiss), and processed with Axiovision 4.5 (Zeiss) and Adobe Photoshop software. Quantifications for the longitudinal sections were performed by counting at least 800 ommatidia from four or more individual flies per genotype, and only sections that include both R7 and R8 layers were counted. Quantifications for the tangential sections use one section for each retina to avoid repeatedly counting the same ommatidia. Retinas for quantifying the whole mount staining are from at least three flies per genotype.

### Polyclonal antibody production

Polyclonal antiserum against Rh6 was generated against a KLH-conjugated peptide from the Rh6 deduced amino acid sequence (CLACGKDDLTSDSRTQAT corresponding to amino acids 344–361). Peptide synthesis, KLH-conjugation, rabbit immunizations and bleeds were performed by GenScript (Piscataway, NJ).

## Supporting information

S1 Text A list of *Drosophila* genotypes used in this study. (DOC)

S1 Fig R8 subtype phenotype enhancement in double and triple heterozygotes with Hippo pathway mutations. (A-I) Cryosections of adult eyes immunostained for Rh5 (red) and Rh6 (green) in control, heterozygous, double heterozygous and triple heterozygous flies with Hippo pathway mutations. Compared to control and heterozygous mutations (A-E), double heterozygous Hippo pathway mutations slightly increased Rh5-expressing R8s and reduced Rh6-expressing R8s (F-H). In flies with triple heterozygous Hippo pathway mutations, the number of the Rh5-expressing R8s were dramatically increased at the expense of the Rh6-expressing R8s (I). See Fig 1 for the quantification of R8 subtypes in these genotypes.
(TIF)

S2 Fig Diagram of the phenotype enhancement screening using the sensitized *wts*^*Zn*^*-kib*^*1*^ flies. (A) Diagram of deficiencies on the third chromosome (from 61B1 to 88C9, 112 deficiencies from the Bloomington Deficiency Kit in this area) that are used in the R8 subtype determinant screening. (B) Diagram showing the fly crossing strategy in the screening.
(TIF)

S3 Fig Pyd is required to specify R8 photoreceptor subtypes. (A-E) Adult eye cryosections immunostained for Rh5 (red) and Rh6 (green) in control, *pyd* mutant or *pyd* knock-down eyes. (A) Control, (B) *pyd*^*B12*^/*pyd*^*B12*^, (C) *pyd*^*B12*^/*pyd*^*J4*^, (D) *lGMR>pyd*^*RNAi*-*HMS00263*^ and (E) *lGMR>pyd*^*RNAi-#450*^. (F) Adult eye tangential cryosection immunostained for Rh5 (red) and Rh6 (green) in *pyd* LOF (*pyd*^*ex180*^/*pyd*^*B12*^) flies. (G) Quantification of R8 subtypes in *pyd* mutants and *pyd* knock-down eyes. Error bars represent standard deviation. NS: not significant. ** p < 0.001. *lGMR-GAL4*: n = 10 retinas, n = 1822 R8s; *pyd*^*B12*^*/pyd*^*B12*^: n = 4 retinas, n = 820 R8s; *pyd*^*B12*^*/pyd*^*J4*^: n = 5 retinas, n = 909 R8s; *lGMR>pyd*^*RNAi*-*HMS00263*^: n = 5 retinas, n = 840 R8s; *lGMR>pyd*^*RNAi-#450*^: n = 5 retinas, n = 806 R8s. (H-I) Retina whole mount staining for control (I) and *pyd* LOF (*pyd*^*ex180*^/*pyd*^*B12*^) eyes. (J) Quantification of R8 subtypes in control and *pyd* LOF eyes (the retina whole mount staining). ** p < 0.001. Control: n = 3 retinas, n = 725 R8s; *pyd*^*ex180*^*/pyd*^*B12*^: n = 3 retinas, n = 524 R8s.
(TIF)

S4 Fig Knock-down of *sd* suppresses *pyd* mutant phenotype. **(Related to Fig 3).** (A-B) Adult eye cryosections immunostained for Rh5 (red) and Rh6 (green) in knock-down of *sd* (A, *otd-GAL4>UAS-sd*^*RNAi*^) and knock-down of *sd* in *pyd* LOF (B, *otd-GAL4>UAS-sd*^*RNAi*^, *pyd*^*ex180*^/*pyd*^*B12*^) flies.
(TIF)

S5 Fig Loss of function of *melt* suppresses *pyd* mutant phenotype. **(Related to Fig 5).** (A-C) Adult eye cryosections immunostained for Rh5 (red) and Rh6 (green) in knock-down of *melt* (A, *otd-GAL4>UAS-melt*^*RNAi*^), *pyd* LOF (B, *pyd*^*ex180*^/*pyd*^*B12*^), and knock-down of *melt* in *pyd* LOF (C, *otd-GAL4>UAS-melt*^*RNAi*^, *pyd*^*ex180*^/*pyd*^*B12*^) flies. (D-F) Adult eye tangential cryosections immunostained for Rh5 (red) and Rh6 (green) in *melt* mutant (D, *melt*^*Δ1*^), *pyd* knock-down (E, *otd-GAL4>UAS*-*pyd*^*RANi-KK*^), and knock-down of *pyd* in *melt* mutant (F, *melt*^*Δ1*^, *otd-GAL4>UAS-pyd*^*RNAi-KK*^) flies.
(TIF)

S6 Fig Misexpression of *su(dx)* induces pR8 subtype fate at the expense of yR8. (A-B) Adult eye cryosections immunostained for Rh5 (red) and Rh6 (green) in control (A) and *su(dx)* misexpression eyes. (A) Control (*lGMR-GAL4*), (B) *su(dx)* misexpression (*lGMR-GAL4*>*su(dx)*). (C) Quantification of R8 subtypes in *su(dx)* misexpression eyes. Error bars represent standard deviation. ** p < 0.001. *lGMR-GAL4*: n = 10 retinas, n = 1822 R8s; *lGMR>su(dx)*: n = 6 retinas, n = 816 R8s.
(TIF)

S7 Fig Kib is not required for the overexpressed Pyd to promote yR8 and suppress pR8 fate specification. **(Related to Fig 7).** (A-C) Adult eye cryosections immunostained for Rh5 (red) and Rh6 (green) in *kib* knock-down (A, *GMR>kib*^*RNAi-HMC03256*^+*Luc*), *pyd* overexpression (B, *GMR>pyd+Luc*) and *kib* knock-down in *pyd* overexpression (C, *GMR>pyd*+*kib*^*RNAi-HMC03256*^) flies. *UAS-Luciferase* (*UAS-Luc*) was used as a control to balance the number of UAS sites. (D) Quantification of the Rh5- and Rh6-expressing R8s in the eyes with the indicated genotypes. NS: not significant. Error bars represent standard deviation. *GMR>kib*^*RNAi-HMC03256*^*+Luc*: n = 4 retinas, n = 901 R8s; *GMR>pyd+Luc*: n = 6 retinas, n = 912 R8s. *GMR>pyd+kib*^*RNAi-HMC03256*^*+Luc*: n = 4 retinas, n = 921 R8s.
(TIF)
