# Supplementary material for: The ZO-1 protein Polychaetoid as an upstream regulator of the Hippo pathway in Drosophila
Source: PLoS Genet. 2021 Nov 8;17(11):e1009894. doi: 10.1371/journal.pgen.1009894 (PMC8610254; doi:10.1371/journal.pgen.1009894)
Supplement: S1 Text — (DOC) [file pgen.1009894.s001.doc]

**S1 Text**

**A list of the genotypes of flies used in this study:**

*pWIZ-wΔ13* was used to knock down the *white* gene and reduce the background fluorescence. *UAS-Luciferase* (*UAS-Luc*) was used to balance the number of UAS sites. *UAS-Dicer2* was used to increase RNAi efficiency. *Cyo*, *TM2*, and *TM6B* are chromosome balancers. *sp* is a 2nd chromosome marker.

**Fig 1:**

Fig 1D: *pWIZ-wΔ13/y^1^w^67c23^; +/Cyo; TM2/TM6B*

Fig 1E: *pWIZ-wΔ13/y^1^w^67c23^; +/Cyo; wts^Zn^-kib^1^/TM2*

Fig 1F: *pWIZ-wΔ13/mer^3^; +/Cyo; wts^Zn^/+*

Fig 1G: *pWIZ-wΔ13/mer^3^; +/Cyo; wts^Zn^-kib^1^/+*

Fig 1H: *control*: *pWIZ-wΔ13/y^1^w^67c23^; +/Cyo; TM2/TM6B*

*wts^Zn^/+*: *pWIZ-wΔ13/y^1^w^67c23^; +/Cyo; wts^Zn^/TM2*

*kib^1^/+*: *pWIZ-wΔ13/y^1^w^67c23^; +/Cyo; kib^1^/+*

*sav^df^/+*: *pWIZ-wΔ13/y^1^w^67c23^; +/Cyo; Df(3R)BSC803/+*

*mer^3^/+*: *pWIZ-wΔ13/mer^3^; +/Cyo; TM2/+*

*wts^Zn^-kib^1^/+*:  *pWIZ-wΔ13//y^1^w^67c23^; +/Cyo; wts^Zn^-kib^1^/TM2*

*wts^Zn^/sav^df^*: *pWIZ-wΔ13/w^1118^; +/Cyo; wts^Zn^/Df(3R)BSC803*

*kib^1^/sav^df^*: *pWIZ-wΔ13/w^1118^; +/Cyo; kib^1^/Df(3R)BSC803*

*wts^Zn^/mer^3^*: *pWIZ-wΔ13/ mer^3^; +/Cyo; wts^Zn^/+*

*mer^3^/kib^1^*: *pWIZ-wΔ13/ mer^3^; +/Cyo; kib^1^/+*

*wts^Zn^-kib^1^/mer^3^*: *pWIZ-wΔ13/ mer^3^; +/Cyo wts^Zn^-kib^1^/+*

*wts^Zn^-kib^1^/sav^df^*: *pWIZ-wΔ13/w^1118^; +/Cyo; wts^Zn^-kib^1^/Df(3R)BSC803*

**Fig 2:**

Fig 2B: *pWIZ-wΔ13/w^1118^; +/Cyo; wts^Zn^-kib^1^/Df(3R)BSC466*

Fig 2C: *pWIZ-wΔ13/w^1118^; +/Cyo; wts^Zn^-kib^1^/Df(3R)ED5330*

Fig 2D*: pWIZ-wΔ13/w^1118^; +/Cyo; wts^Zn^-kib^1^/Df(3R)pyd^B12^*

Fig 2E: *Df(3R)BSC466*: *pWIZ-wΔ13/w^1118^; +/Cyo; wts^Zn^-kib^1^/Df(3R)BSC466*

*Df(3R)ED5330: pWIZ-wΔ13/w^1118^; +/Cyo; wts^Zn^-kib^1^/Df(3R)ED5330*

*Df(3R)Exel6150: pWIZ-wΔ13/w^1118^; +/Cyo; wts^Zn^-kib^1^/Df(3R)Exel6150*

*Df(3R)BSC478: pWIZ-wΔ13/w^1118^; +/Cyo; wts^Zn^-kib^1^/Df(3R)BSC478*

*Df(3R)BSC506: pWIZ-wΔ13/w^1118^; +/Cyo; wts^Zn^-kib^1^/Df(3R)BSC506*

*Df(3R)pyd^B12^*: *pWIZ-wΔ13/w^1118^; +/Cyo; wts^Zn^-kib^1^/Df(3R)pyd^B12^*

*Df(3R)BSC666: pWIZ-wΔ13/w^1118^; +/Cyo; wts^Zn^-kib^1^/Df(3R)BSC666*

*Df(3R)Exel6152: pWIZ-wΔ13/w^1118^; +/Cyo; wts^Zn^-kib^1^/Df(3R)Exel6152*

Fig 2F: *pWIZ-wΔ13/y^1^w^67c23^; +/Cyo; pyd^ex180^/pyd^ex180^*

Fig 2G*: pWIZ-wΔ13/y^1^w^67c23^; +/Cyo; pyd^ex147^/pyd^ex147^*

Fig 2H: *pyd^ex180^*: *pWIZ-wΔ13//y^1^w^67c23^; +/Cyo; pyd^ex180^/pyd^ex180^*

*pyd^ex147^*: *pWIZ-wΔ13/y^1^w^67c23^; +/Cyo; pyd^ex147^/pyd^ex147^*

**Fig 3:**

Fig 3A: *y^1^w^67c23^; lGMR-GAL4,pWIZ-wΔ13,UAS-Dicers/Cyo; TM2/TM6B*

Fig 3B: *y^1^w^67c23^; lGMR-GAL4,pWIZ-wΔ13,UAS-Dicers/Cyo; pyd^RNAi-kk105581^/TM2*

Fig 3C: *pWIZ-wΔ13/y^1^w^67c23^; sens-GLA4/Cyo; Sens-GAL4/TM2*

Fig 3D: *pWIZ-wΔ13/y^1^w^67c23^; sens-GLA4/Cyo; Sens-GAL4/ pyd^RNAi-kk105581^*

Fig 3E: *pWIZ-wΔ13/y^1^w^67c23^; sev-GLA4/Cyo; TM2/TM6B*

Fig 3F: *pWIZ-wΔ13/y^1^w^67c23^; sev-GLA4/Cyo; pyd^RNAi-kk105581^/TM2*

Fig 3G: *sev^14^; lGMR-GAL4,pWIZ-wΔ13,UAS-Dicers/Cyo; TM2/TM6B*

Fig 3H: *sev^14^; lGMR-GAL4,pWIZ-wΔ13,UAS-Dicers/Cyo; pyd^RNAi-kk105581^/TM2*

Fig 3I: *pWIZ-wΔ13/y^1^w^67c23^; lGMR-GLA4/yki^RNAi-kk109756^; TM2/TM6B*

Fig 3J: *pWIZ-wΔ13/y^1^w^67c23^; lGMR-GLA4/yki^RNAi-kk109756^; pyd^ex180^/Df(3R)pyd^B12^*

Fig K: *sd^ΔB^-FRT19A/FRT19A-GMR-Hid*; *pWIZ-wΔ13/eyeless-flippase; TM2/TM6B*

Fig 3L: *sd^ΔB^-FRT19A/FRT19A-GMR-Hid*; *pWIZ-wΔ13/eyeless-flippase;*

*pyd^ex180^/Df(3R)pyd^B12^*

**Fig 4:**

Fig 4A: *pWIZ-wΔ13/y^1^w^67c23^; sens-GLA4/Cyo; wts^Zn^/TM2*

Fig 4B: *pWIZ-wΔ13/y^1^w^67c23^; sens-GLA4/ pyd^RNAi-kk105581^; wts^Zn^/TM2*

Fig 4C: *pWIZ-wΔ13/y^1^w^67c23^; sens-GLA4/Cyo; melt450-nLacZ/TM2*

Fig 4D: *pWIZ-wΔ13/y^1^w^67c23^; sens-GLA4/ pyd^RNAi-kk105581^; melt450-nLacZ/TM2*

Fig 4G: *pWIZ-wΔ13/y^1^w^67c23^; UAS-nGFP/Cyo; p[GawB]^NP4419^/TM2*

Fig 4H: *pWIZ-wΔ13/UAS-melt; UAS-nGFP/otd-GAL4; p[GawB]^NP4419^/TM2*

**Fig 5:**

Fig 5A: *pWIZ-wΔ13/y^1^w^67c23^; +/Cyo; pyd^ex180^/Df(3R)pyd^B12^*

Fig 5B: *pWIZ-wΔ13/y^1^w^67c23^; lGMR-GAL4/UAS-wts; TM2/TM6B*

Fig 5C: *pWIZ-wΔ13/y^1^w^67c23^; lGMR-GAL4/UAS-wts; pyd^ex180^/Df(3R)pyd^B12^*

Fig 5D: *pWIZ-wΔ13/y^1^w^67c23^; lGMR-GAL4/UAS-hpo; pyd^ex180^/Df(3R)pyd^B12^*

Fig 5E: *pWIZ-wΔ13/y^1^w^67c23^; GMR-sav/Cyo; TM2/TM6B*

Fig 5F: *pWIZ-wΔ13/y^1^w^67c23^; GMR-sav/Cyo; pyd^ex180^/Df(3R)pyd^B12^*

Fig 5G: *pWIZ-wΔ13/y^1^w^67c23^; lGMR-GAL4/UAS-melt^RNAi-kk101301^; TM2/TM6B*

Fig 5H: *pWIZ-wΔ13/y^1^w^67c23^; lGMR-GAL4/UAS-melt^RNAi-kk101301^; pyd^ex180^/Df(3R)pyd^B12^*

Fig 5J: *pWIZ-wΔ13/y^1^w^67c23^; GMR-GAL4/Cyo; TM2/TM6B*

Fig 5K*: pWIZ-wΔ13/y^1^w^67c23^; GMR-GAL4/Cyo; UAS-GFP-pyd/UAS-Luc*

Fig 5L*: pWIZ-wΔ13/y^1^w^67c23^; GMR-GAL4/wts^RNAi-kk101055^*; *UAS-GFP-pyd/TM2*

Fig 5M*: pWIZ-wΔ13/y^1^w^67c23^; GMR-GAL4/hpo^RNAi-kk101704^*; *UAS-GFP-pyd/TM2*

Fig 5N*: pWIZ-wΔ13/y^1^w^67c23^; GMR-GAL4/sav^RNAi-kk107562^*; *UAS-GFP-pyd/TM2*

Fig 5O*: pWIZ-wΔ13/y^1^w^67c23^; GMR-GAL4/mats^RNAi-kk100140^*; *UAS-GFP-pyd/TM2*

**Fig 6:**

Fig 6A: *pWIZ-wΔ13/y^1^w^67c23^; lGMR-GAL4/Cyo; TM2/TM6B*

Fig 6B: *pWIZ-wΔ13/y^1^w^67c23^; +/Cyo; pez^1^/pez^2^*

Fig 6C: *pWIZ-wΔ13/y^1^w^67c23^; lGMR-GAL4/Cyo; UAS-pez/TM2*

Fig 6D: *pWIZ-wΔ13/y^1^w^67c23^; lGMR-GAL4/Cyo; UAS-pez, pyd^ex180^/Df(3R)pyd^B12^*

Fig 6E: *pWIZ-wΔ13/y^1^w^67c23^; GMR-GAL4/Cyo; UAS-pez^RNAi-HMS00862^/UAS-Luc*

Fig 6F: *pWIZ-wΔ13/y^1^w^67c23^; GMR-GAL4/Cyo; UAS-pez^RNAi-HMS00862^/UAS-GFP-pyd*

Fig 6H: *y^1^w^67c23^; lGMR-GAL4,pWIZ-wΔ13,UAS-Dicers/su(dx)^RNAi-HMS05478^; TM2/+*

Fig 6I: *y^1^w^67c23^; su(dx)2/su(dx)^32^; TM2/TM6B*

Fig 6J: *pWIZ-wΔ13/y^1^w^67c23^; +/Cyo; pyd^ex180^/Df(3R)pyd^B12^*

Fig 6K: *y^1^w^67c23^; lGMR-GAL4,pWIZ-wΔ13,UAS-Dicers/su(dx)^RNAi-HMS05478^;*

*pyd^ex180^/Df(3R)pyd^B12^*

Fig 6L: *pWIZ-wΔ13/y^1^w^67c23^; GMR-GAL4/UAS-su(dx); UAS-Luc/TM2*

Fig 6M: *pWIZ-wΔ13/y^1^w^67c23^; GMR-GAL4/UAS-su(dx); UAS-GFP-pyd/TM2*

**Fig 7:**

Fig 7A: *pWIZ-wΔ13/y^1^w^67c23^; +/Cyo; pyd^ex180^/Df(3R)pyd^B12^*

Fig 7B: *pWIZ-wΔ13/y^1^w^67c23^; lGMR-GAL4/UAS-kib; TM2/TM6B*

Fig 7C: *pWIZ-wΔ13/y^1^w^67c23^; lGMR-GAL4/UAS-kib; pyd^ex180^/Df(3R)pyd^B12^*

Fig 7D: *pWIZ-wΔ13/y^1^w^67c23^; GMR-GAL4/Cyo; UAS-GFP-pyd/UAS-Luc*

Fig 7E: *pWIZ-wΔ13/y^1^w^67c23^; GMR-GAL4/kib^RNAi-KK108510^; UAS-Luc/TM2*

Fig 7F: *pWIZ-wΔ13/y^1^w^67c23^; GMR-GAL4/kib^RNAi-KK108510^; UAS-GFP-pyd/TM2*

Fig 7G: *pWIZ-wΔ13/y^1^w^67c23^; lGMR-GAL4/UAS-su(dx); UAS-Luc/TM2*

Fig 7H: *pWIZ-wΔ13/y^1^w^67c23^; lGMR-GAL4/UAS-su(dx); UAS-kib/TM2*

**S1 Fig:**

S1A Fig: *pWIZ-wΔ13//y^1^w^67c23^; +/Cyo; TM2/TM6B*

S1B Fig: *pWIZ-wΔ13/y^1^w^67c23^; +/Cyo; wts^Zn^/TM2*

S1C Fig: *pWIZ-wΔ13/y^1^w^67c23^; +/Cyo; kib^1^/+*

S1D Fig: *pWIZ-wΔ13/y^1^w^67c23^; +/Cyo; Df(3R)BSC803/+*

S1E Fig: *pWIZ-wΔ13/mer^3^; +/Cyo; TM2/+*

S1F Fig: *pWIZ-wΔ13/mer^3^; +/Cyo; kib^1^/+*

S1G Fig: *pWIZ-wΔ13/w^1118^; +/Cyo; kib^1^/Df(3R)BSC803*

S1H Fig: *pWIZ-wΔ13/w^1118^; +/Cyo; wts^Zn^/Df(3R)BSC803*

S1I Fig:  *pWIZ-wΔ13/w^1118^; +/Cyo wts^Zn^-kib^1^/Df(3R)BSC803*

**S3 Fig:**

S3A Fig:  *y^1^w^67c23^; longGMR-GAL4, UAS-Dicer2, pWIZ-wΔ13 /Cyo; Tm2/TM6B*

S3B Fig: *pWIZ-wΔ13/y^1^w^67c23^ +/Cyo; Df(3R)pyd^B12^/Df(3R)pyd^B12^*

S3C Fig: *pWIZ-wΔ13/y^1^w^67c23^; +/Cyo; Df(3R)pyd^B12^/pyd^J4^*

S3D Fig: *y^1^w^67c23^; longGMR-GAL4, UAS-Dicer2, pWIZ-wΔ13/Cyo; pyd^RNAi-HMS00263^/TM2*

S3E Fig:  *y^1^w^67c23^; longGMR-GAL4, UAS-Dicer2, pWIZ-wΔ13/Cyo; pyd^RNAi-#450^/TM2*

S3F Fig: *pWIZ-wΔ13/y^1^w^67c23^ +/Cyo; pyd^ex180^/Df(3R)pyd^B12^*

S3I Fig: *pWIZ-wΔ13/y^1^w^67c23^*; *sp/Cyo; TM2/TM6B*

S3J Fig: *pWIZ-wΔ13/y^1^w^67c23^*; *+/Cyo; pyd^ex180^/Df(3R)pyd^B12^*

**S4 Fig:**

S4A Fig: *pWIZ-wΔ13/y^1^w^67c23^; otd-GAL4/ sd^RNAi-kk108877^*; *TM2/TM6B*

S4B Fig: *pWIZ-wΔ13/y^1^w^67c23^; otd-GAL4/ sd^RNAi-kk108877^*; *pyd^ex180^/Df(3R)pyd^B12^*

**S5 Fig**:

S5A Fig: *pWIZ-wΔ13/y^1^w^67c23^; otd-GAL4/melt^RNAi-kk101301^*; *TM2/TM6B*

S5B Fig: *pWIZ-wΔ13/y^1^w^67c23^*; *+/Cyo; pyd^ex180^/Df(3R)pyd^B12^*

S5C Fig: *pWIZ-wΔ13/y^1^w^67c23^*; *otd-GAL4/melt^RNAi-kk101301^*; *pyd^ex180^/Df(3R)pyd^B12^*

S5D Fig: *melt^Δ1^/melt^Δ1^*; *otd-GAL4, pWIZ-wΔ13/ Cyo*; *TM2/TM6B*

S5E Fig: *pWIZ-wΔ13/y^1^w^67c23^*; *otd-GAL4, pWIZ-wΔ13/ pyd^RNAi-kk105581^*; *TM2/TM6B*

S5F Fig: *melt^Δ1^/melt^Δ1^*; *otd-GAL4, pWIZ-wΔ13/ pyd^RNAi-kk105581^*; *TM2/TM6B*

**S6 Fig:**

S6A Fig: *pWIZ-wΔ13/y^1^w^67c23^; lGMR-GAL4/Cyo; TM2/TM6B*

S6B Fig: *pWIZ-wΔ13/y^1^w^67c23^; lGMR-GAL4/UAS-su(dx); TM2/TM6B*

**S7 Fig:**

S7A Fig: *pWIZ-wΔ13/y^1^w^67c23^; GMR-GAL4/kib^RNAi-HMC03256^; UAS-Luc/TM2*

S7B Fig: *pWIZ-wΔ13/y^1^w^67c23^; GMR-GAL4/Cyo; UAS-GFP-pyd/UAS-Luc*

S7C Fig: *pWIZ-wΔ13/y^1^w^67c23^; GMR-GAL4/kib^RNAi-HMC03256^; UAS-GFP-pyd/TM2*
