# Supplementary figures and images for: The ZO-1 protein Polychaetoid as an upstream regulator of the Hippo pathway in Drosophila
Source: PLoS Genet. 2021 Nov 8;17(11):e1009894. doi: 10.1371/journal.pgen.1009894 (PMC8610254; doi:10.1371/journal.pgen.1009894)

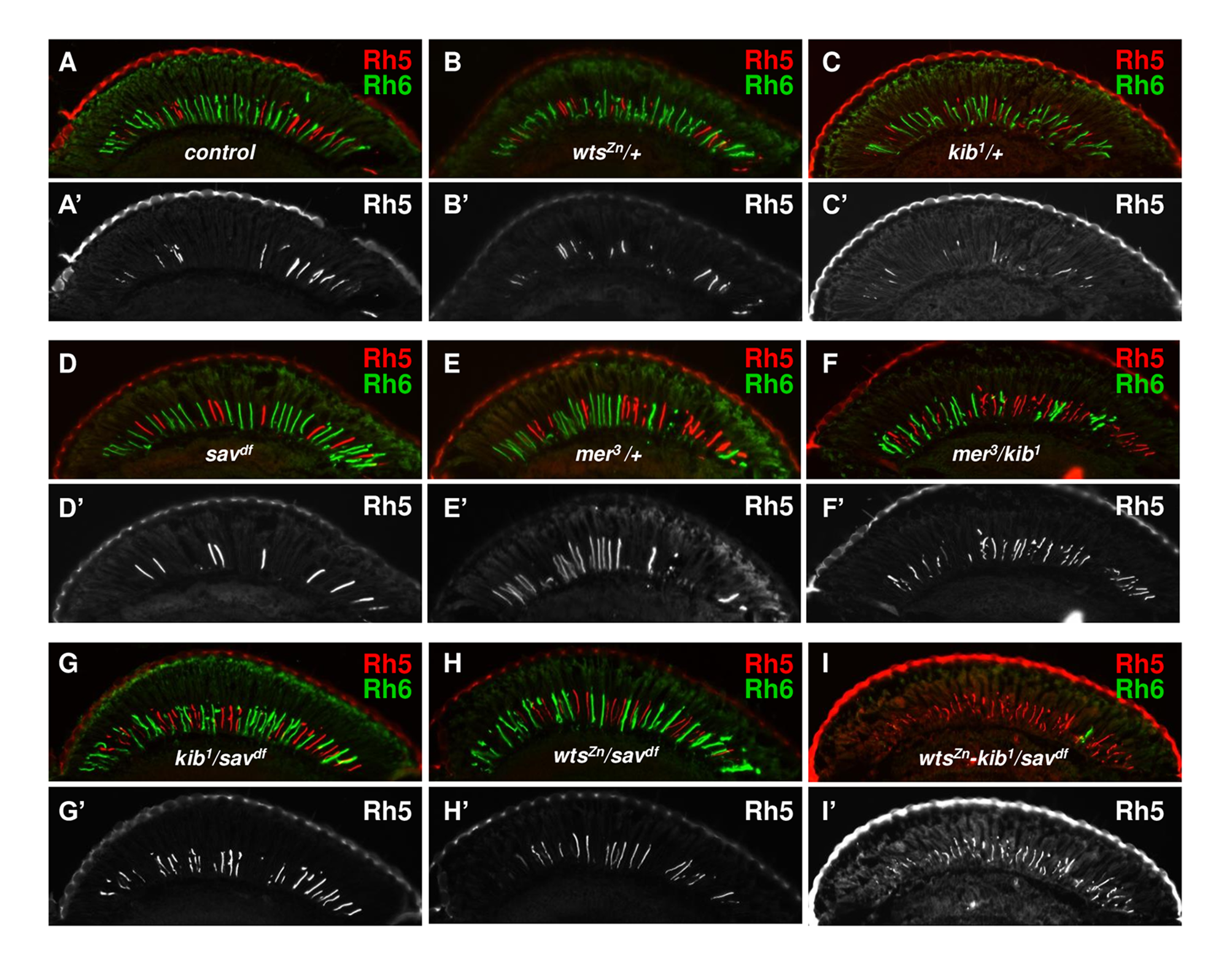

Supplement: S1 Fig — (A-I) Cryosections of adult eyes immunostained for Rh5 (red) and Rh6 (green) in control, heterozygous, double heterozygous and triple heterozygous flies with Hippo pathway mutations. Compared to control and heterozygous mutations (A-E), double heterozygous Hippo pathway mutations slightly increased Rh5-expressing R8s and reduced Rh6-expressing R8s (F-H). In flies with triple heterozygous Hippo pathway mutations, the number of the Rh5-expressing R8s were dramatically increased at the expense of the Rh6-expressing R8s (I). See Fig 1 for the quantification of R8 subtypes in these genotypes. (TIF) [file pgen.1009894.s002.tif]

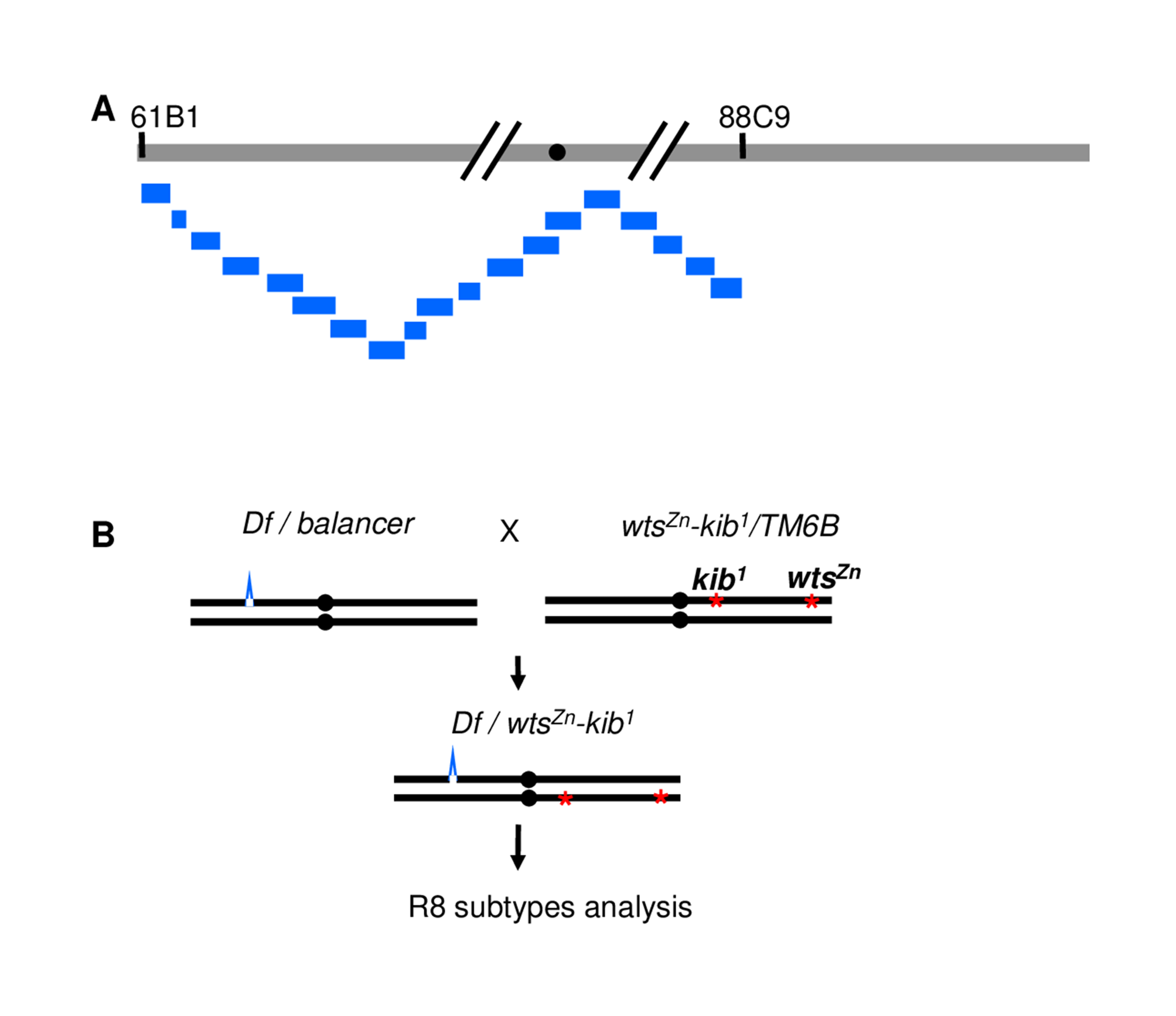

Supplement: S2 Fig — (A) Diagram of deficiencies on the third chromosome (from 61B1 to 88C9, 112 deficiencies from the Bloomington Deficiency Kit in this area) that are used in the R8 subtype determinant screening. (B) Diagram showing the fly crossing strategy in the screening. (TIF) [file pgen.1009894.s003.tif]

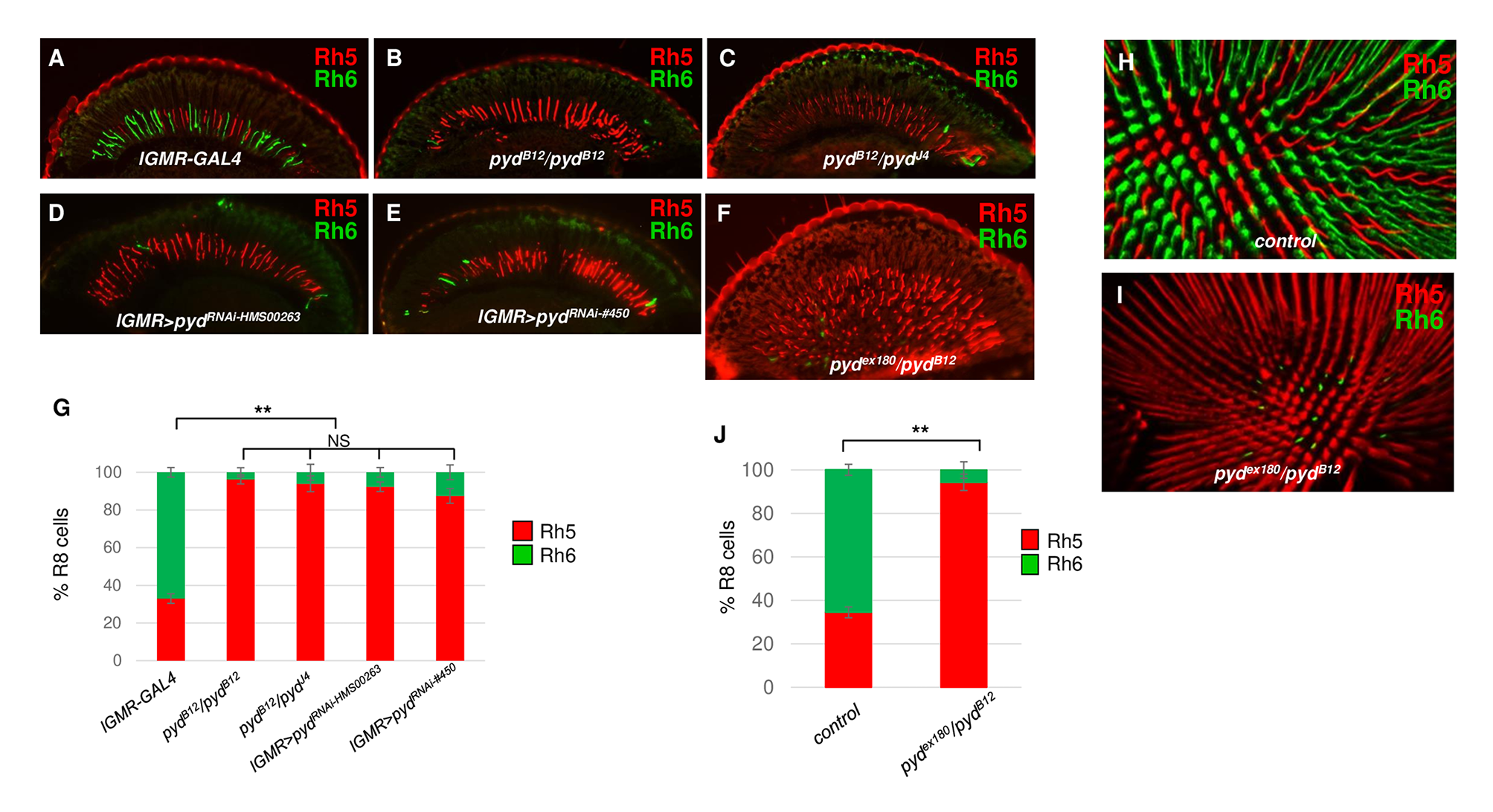

Supplement: S3 Fig — (A-E) Adult eye cryosections immunostained for Rh5 (red) and Rh6 (green) in control, pyd mutant or pyd knock-down eyes. (A) Control, (B) pydB12/pydB12, (C) pydB12/pydJ4, (D) lGMR>pydRNAi-HMS00263 and (E) lGMR>pydRNAi-#450. (F) Adult eye tangential cryosection immunostained for Rh5 (red) and Rh6 (green) in pyd LOF (pydex180/pydB12) flies. (G) Quantification of R8 subtypes in pyd mutants and pyd knock-down eyes. Error bars represent standard deviation. NS: not significant. ** p < 0.001. lGMR-GAL4: n = 10 retinas, n = 1822 R8s; pydB12/pydB12: n = 4 retinas, n = 820 R8s; pydB12/pydJ4: n = 5 retinas, n = 909 R8s; lGMR>pydRNAi-HMS00263: n = 5 retinas, n = 840 R8s; lGMR>pydRNAi-#450: n = 5 retinas, n = 806 R8s. (H-I) Retina whole mount staining for control (I) and pyd LOF (pydex180/pydB12) eyes. (J) Quantification of R8 subtypes in control and pyd LOF eyes (the retina whole mount staining). ** p < 0.001. Control: n = 3 retinas, n = 725 R8s; pydex180/pydB12: n = 3 retinas, n = 524 R8s. (TIF) [file pgen.1009894.s004.tif]

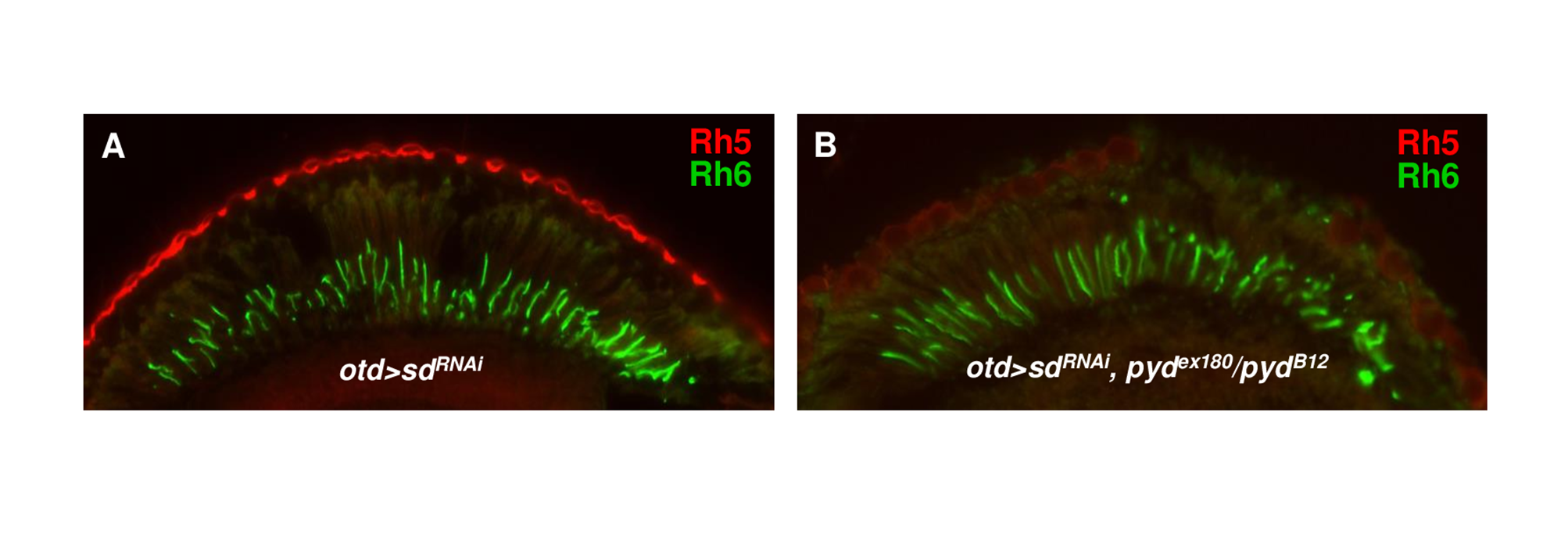

Supplement: S4 Fig — (Related to Fig 3). (A-B) Adult eye cryosections immunostained for Rh5 (red) and Rh6 (green) in knock-down of sd (A, otd-GAL4>UAS-sdRNAi) and knock-down of sd in pyd LOF (B, otd-GAL4>UAS-sdRNAi, pydex180/pydB12) flies. (TIF) [file pgen.1009894.s005.tif]

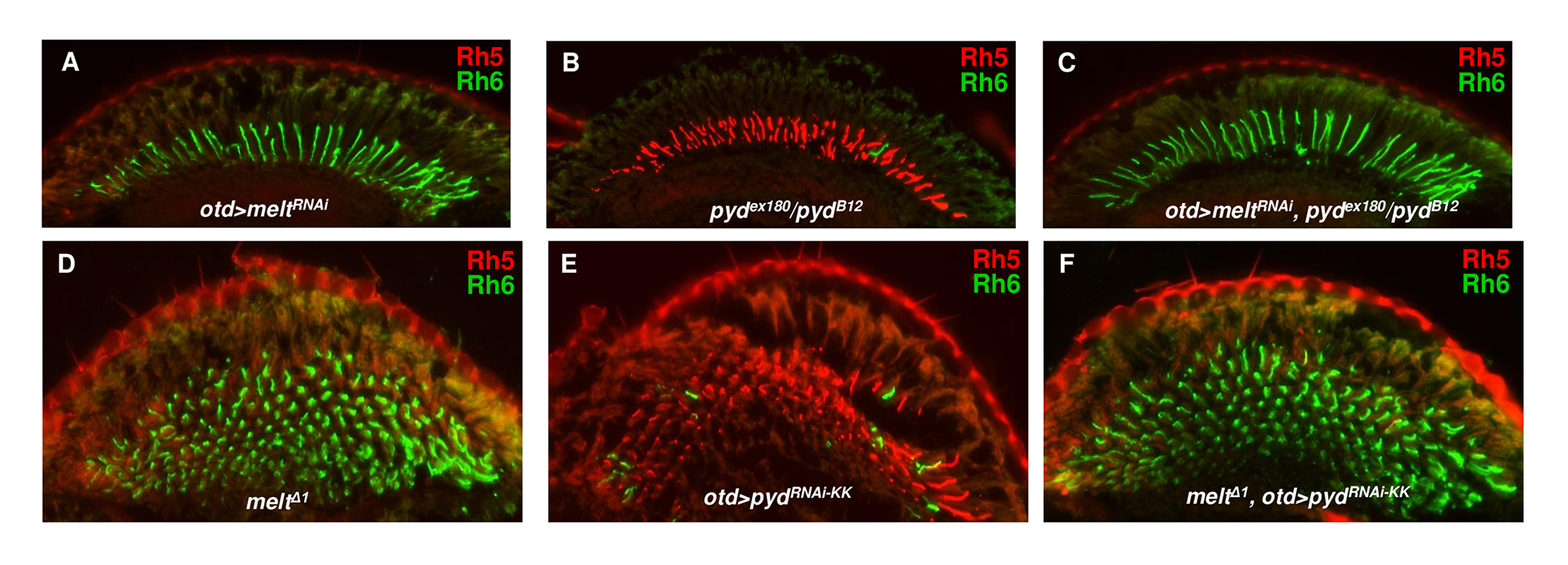

Supplement: S5 Fig — (Related to Fig 5). (A-C) Adult eye cryosections immunostained for Rh5 (red) and Rh6 (green) in knock-down of melt (A, otd-GAL4>UAS-meltRNAi), pyd LOF (B, pydex180/pydB12), and knock-down of melt in pyd LOF (C, otd-GAL4>UAS-meltRNAi, pydex180/pydB12) flies. (D-F) Adult eye tangential cryosections immunostained for Rh5 (red) and Rh6 (green) in melt mutant (D, meltΔ1), pyd knock-down (E, otd-GAL4>UAS-pydRANi-KK), and knock-down of pyd in melt mutant (F, meltΔ1, otd-GAL4>UAS-pydRNAi-KK) flies. (TIF) [file pgen.1009894.s006.tif]

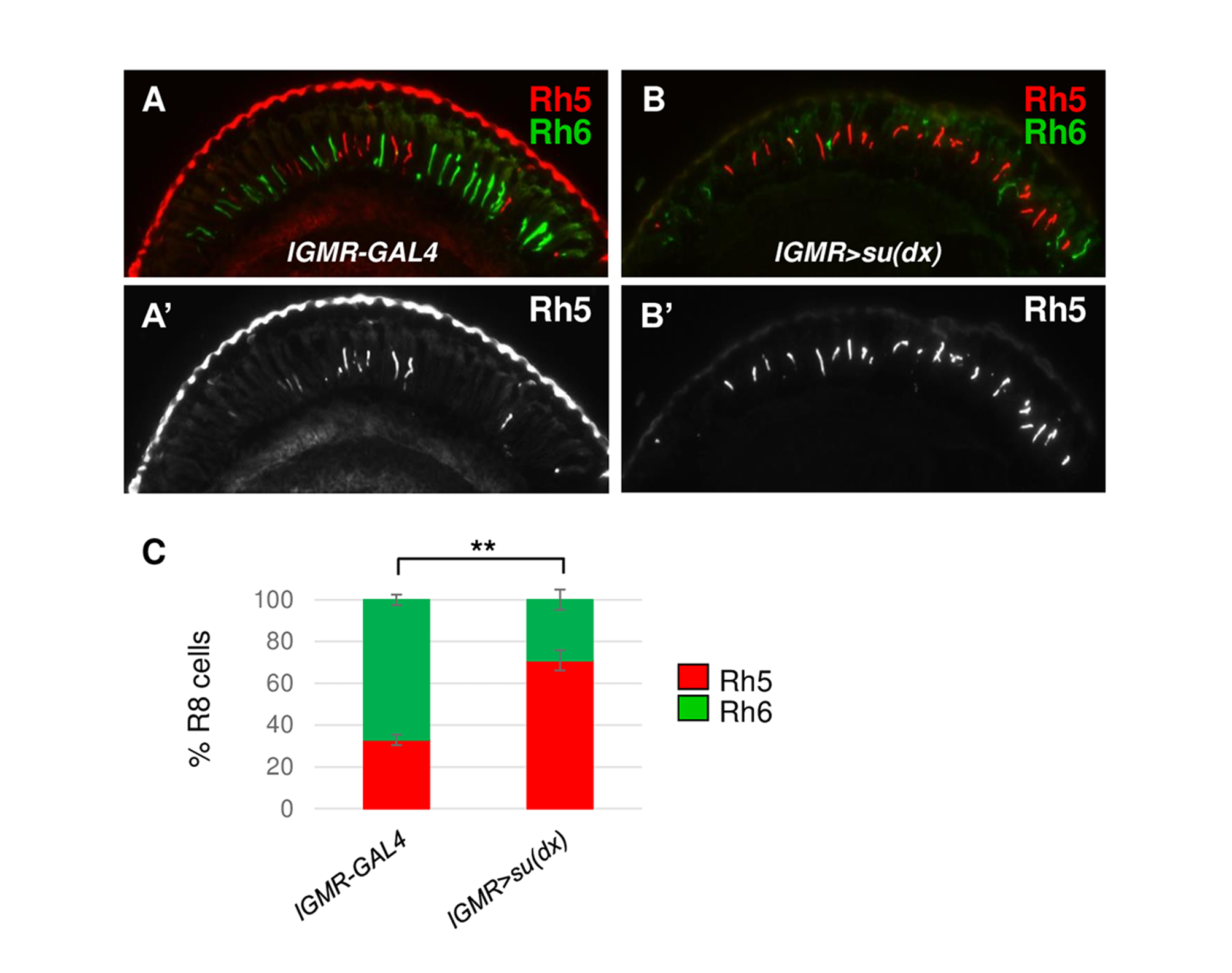

Supplement: S6 Fig — (A-B) Adult eye cryosections immunostained for Rh5 (red) and Rh6 (green) in control (A) and su(dx) misexpression eyes. (A) Control (lGMR-GAL4), (B) su(dx) misexpression (lGMR-GAL4>su(dx)). (C) Quantification of R8 subtypes in su(dx) misexpression eyes. Error bars represent standard deviation. ** p < 0.001. lGMR-GAL4: n = 10 retinas, n = 1822 R8s; lGMR>su(dx): n = 6 retinas, n = 816 R8s. (TIF) [file pgen.1009894.s007.tif]

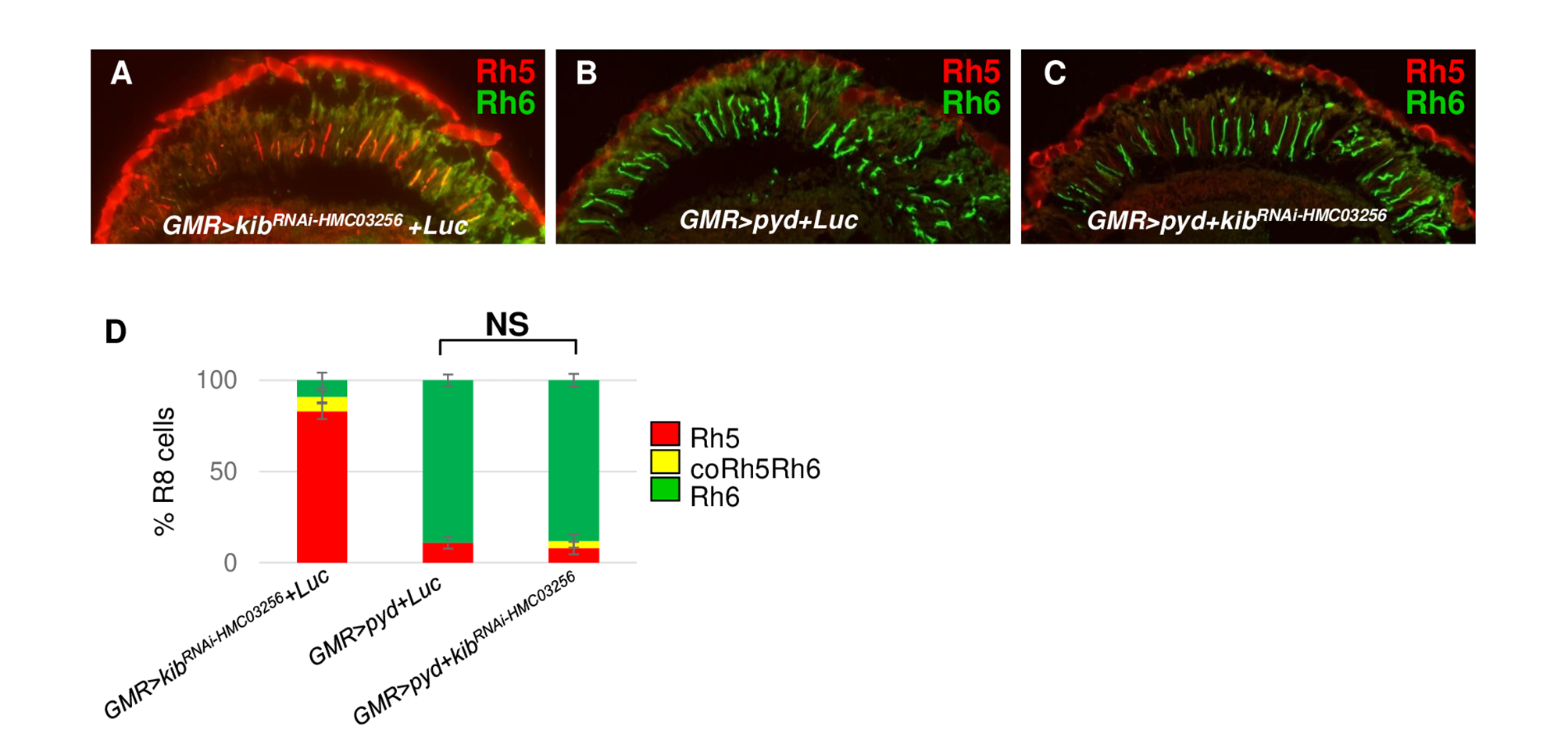

Supplement: S7 Fig — (Related to Fig 7). (A-C) Adult eye cryosections immunostained for Rh5 (red) and Rh6 (green) in kib knock-down (A, GMR>kibRNAi-HMC03256+Luc), pyd overexpression (B, GMR>pyd+Luc) and kib knock-down in pyd overexpression (C, GMR>pyd+kibRNAi-HMC03256) flies. UAS-Luciferase (UAS-Luc) was used as a control to balance the number of UAS sites. (D) Quantification of the Rh5- and Rh6-expressing R8s in the eyes with the indicated genotypes. NS: not significant. Error bars represent standard deviation. GMR>kibRNAi-HMC03256+Luc: n = 4 retinas, n = 901 R8s; GMR>pyd+Luc: n = 6 retinas, n = 912 R8s. GMR>pyd+kibRNAi-HMC03256+Luc: n = 4 retinas, n = 921 R8s. (TIF) [file pgen.1009894.s008.tif]
